# Cdk5 regulates IP3R1-mediated Ca^2+^ dynamics and Ca^2+^-mediated cell proliferation

**DOI:** 10.1007/s00018-022-04515-8

**Published:** 2022-08-24

**Authors:** Saranya NavaneethaKrishnan, Vincent Law, Jungkwon Lee, Jesusa L. Rosales, Ki-Young Lee

**Affiliations:** Department of Cell Biology and Anatomy, Arnie Charbonneau Cancer and Alberta Children’s Hospital Research Institutes, Cumming School of Medicine, University of Calgary, Calgary, AB T2N 4N1 Canada

**Keywords:** Proliferation, Cdk5, Ca^2+^ signaling, IP3R

## Abstract

Loss of cyclin-dependent kinase 5 (Cdk5) in the mitochondria-associated endoplasmic reticulum (ER) membranes (MAMs) increases ER–mitochondria tethering and ER Ca^2+^ transfer to the mitochondria, subsequently increasing mitochondrial Ca^2+^ concentration ([Ca^2+^]_mt_). This suggests a role for Cdk5 in regulating intracellular Ca^2+^ dynamics, but how Cdk5 is involved in this process remains to be explored. Using ex vivo primary mouse embryonic fibroblasts (MEFs) isolated from *Cdk5*^*−/−*^ mouse embryos, we show here that loss of Cdk5 causes an increase in cytosolic Ca^2+^concentration ([Ca^2+^]_cyt_), which is not due to reduced internal Ca^2+^ store capacity or increased Ca^2+^ influx from the extracellular milieu. Instead, by stimulation with ATP that mediates release of Ca^2+^ from internal stores, we determined that the rise in [Ca^2+^]_cyt_ in *Cdk5*^*−/−*^ MEFs is due to increased inositol 1,4,5-trisphosphate receptor (IP3R)-mediated Ca^2+^ release from internal stores. Cdk5 interacts with the IP3R1 Ca^2+^ channel and phosphorylates it at Ser_421_. Such phosphorylation controls IP3R1-mediated Ca^2+^ release as loss of Cdk5, and thus, loss of IP3R1 Ser_421_ phosphorylation triggers an increase in IP3R1-mediated Ca^2+^ release in *Cdk5*^*−/−*^ MEFs, resulting in elevated [Ca^2+^]_cyt_. Elevated [Ca^2+^]_cyt_ in these cells further induces the production of reactive oxygen species (ROS), which upregulates the levels of Nrf2 and its targets, Prx1 and Prx2. *Cdk5*^*−/−*^ MEFs, which have elevated [Ca^2+^]_cyt_, proliferate at a faster rate compared to wt, and *Cdk5*^*−/−*^ embryos have increased body weight and size compared to their wt littermates. Taken together, we show that altered IP3R1-mediated Ca^2+^ dynamics due to Cdk5 loss correspond to accelerated cell proliferation that correlates with increased body weight and size in *Cdk5*^*−/−*^ embryos.

**Supplementary Information:**

The online version contains supplementary material available at 10.1007/s00018-022-04515-8.

## Introduction

Cdk5 belongs to the Cdk family of small serine/threonine kinases, which, together with their respective cyclin activators, regulate the eukaryotic cell cycle [1]. It was identified based on its structural similarity to Cdk1 (Cdc2) and Cdk2 [2–4], but most Cdk5 studies are directed at non-cell cycle events. Nonetheless, there is increasing evidence implicating a role for Cdk5 in cell cycle progression and proliferation [5–8]. For example, in human HeLa cervical epithelial cells, Cdk5 and its activator, p35, have been mapped to centrosomes and suggested to regulate centrosome-mediated cell cycle events [9]. In addition, Cdk5 was found to suppress the neuronal cell cycle [6, 10, 11], particularly at G_1_/S [5, 8, 10], and in non-neuronal cells, Cdk5 was found to be required for intra-S and G_2_/M cell-cycle checkpoints [7]. Cdk5 regulates cell-cycle progression by downregulating p21^*CIP1*^ [1, 12, 13] and p27^*KIP1*^ [1, 13], and cell proliferation through modulation of AKT [13, 14], STAT3 [15, 16] or ERK5 [17]. However, gaps remain in our understanding of how Cdk5 regulates the cell cycle and cell proliferation.

The manner in which the various intracellular Ca^2+^ channels, pumps and exchangers are distributed allows extracellular stimuli to induce [Ca^2+^]_cyt_ oscillations in a highly defined spatial and temporal patterns, inducing specific cellular responses such as cell proliferation [18, 19]. Increases in [Ca^2+^]_cyt_ are triggered through a number of mechanisms, including entry from the extracellular milieu, reduced internal Ca^2+^ store capacity, and Ca^2+^ release from internal stores, primarily the endoplasmic reticulum (ER). The role of Cdk5 in regulating intracellular Ca^2+^ dynamics has been primarily examined in neurons where it is abundantly expressed [3, 20]. For example, neuronal Cdk5 has been implicated in regulating external Ca^2+^ entry from the extracellular milieu. Cdk5 phosphorylates the P/Q-type voltage-dependent Ca^2+^ channel (VDCC), supressing external Ca^2+^ entry [21]. Cdk5 also phosphorylates the N-type VDCC [22] and the transient receptor potential cation channel subfamily V member 1 (TrpV1) [23–26], stimulating Ca^2+^ influx from the extracellular milieu. In nociceptive neurons, Cdk5-mediated phosphorylation of the purinergic P2X receptor 2 (P2X2)’s full-size isoform (P2X2aR) at Thr_372_ stimulates external Ca^2+^ entry [27], whereas P2X3R phosphorylation downregulates external Ca^2+^ influx [28]. Thus, Cdk5 regulation of Ca^2+^ influx from the extracellular milieu is dependent upon its target, indicating the need to understand the cellular context in various experimental model systems [29]. In mesencephalic neurons and NGF-differentiated sympathetic-like neuronal cells, ceramide induces stimulation of Cdk5 activity, which causes tau hyperphosphorylation, leading to the formation of paired helical filaments (PHFs) and subsequently neuronal cell death. Cdk5-mediated tau phosphorylation also causes an increase in Ca^2+^ transfer from the ER to mitochondria through enhancement of ER-mitochondria contacts [30]. In separate studies, using NGF-differentiated sympathetic-like neuronal cells, Choi and Chung investigated Cdk5 regulation of intracellular Ca^2+^ dynamics using the Cdk5 inhibitors, roscovitine (ros, 50 µM) and olomoucine (olo, 100 µM) [31]. However, the concentrations used to inhibit Cdk5 in this study lack specificity as other kinases such as Cdk1 (ros, IC_50_ = 0.65 µM; olo, IC_50_ = 7 µM), Cdk2 (ros, IC_50_ = 0.7 µM; olo, IC_50_ = 7 µM) and ERK1 (ros, IC_50_ = 34 µM; olo, IC_50_ = 25 µM) could have also been inhibited. In non-neuronal cells, the specific role of Cdk5 in regulating intracellular Ca^2+^ dynamics remains unknown.

The IP3R, an ER transmembrane protein that forms a Ca^2+^ channel in its transmembrane domain and an IP3-binding site on its cytosolic face [32], forms the major route for Ca^2+^ release from the ER. When extracellular soluble agonists bind a G protein-coupled receptor, phospholipase C (PLC) is activated, producing IP3 from the hydrolysis of phosphatidylinositol 4,5-bisphosphate (PIP2). IP3 binding to IP3R induces opening of this channel and release of Ca^2+^ from the ER. Ca^2+^ released from the ER is then mobilized to the mitochondria [33] through the voltage-dependent anion channels (VDACs) in the outer mitochondrial membrane (OMM) and the mitochondrial calcium uniporter (MCU) channels in the inner mitochondrial membrane (IMM). In previous studies [34], we demonstrated that loss of Cdk5 in MEFs increases ER–mitochondria tethering and ER Ca^2+^ transfer to the mitochondria, subsequently increasing [Ca^2+^]_mt_. This points to a role for Cdk5 in regulating intracellular Ca^2+^ dynamics. Indeed, Cdk5 localizes in the MAM ER-mitochondria interface [34], and thus is well placed to influence ER Ca^2+^ release through IP3R, which is regulated by IP3R phosphorylation [18]. Cdk5 has a preferred phosphorylation consensus sequence of (S/T)PX(K/H/R) [3], and among the IP3R isoforms, IP3R1 has two potential Cdk5 phosphorylation sites: S_421_PLK and T_799_PVK [35]. IP3R2 is insensitive to ATP and does not contain possible Cdk5 phosphorylation sites; IP3R3 has Thr_799_ but ATP-induced, IP3R3-mediated Ca^2+^ release is much less significant than that mediated by IP3R1 [36]. It is possible that Cdk5 interacts with and phosphorylates IP3R1, regulating its channel opening. In fact, the Cdk5-related kinase, Cdk1 phosphorylates IP3R1 at Thr_799_ causing IP3R1 opening [35, 37]. Conversely, ERK1/2 phosphorylation of IP3R1 at Ser_436_ decreases IP3 binding and thus IP3-induced Ca^2+^ release [38–40].

Interplay between [Ca^2+^]_cyt_ and ROS signaling has been reported [41, 42]. Cellular ROS are metabolic byproducts and act as secondary messengers in signaling pathways at sub-toxic levels. Oxidative stress, however, activates the nuclear factor erythroid 2-related factor 2 (Nrf2) transcription factor by inhibiting its negative regulator, Keap1. Nuclear translocation of activated Nrf2 results in the production of the antioxidant enzymes, peroxiredoxins (Prx1 and Prx2), catalase, glutathione peroxidase (GPX), and heme oxygenase-1 (HO-1), to maintain optimal cellular redox balance [43].

In this study, we utilized the *Cdk5*^−/−^ mouse model and corresponding ex vivo MEFs to explore the mechanisms by which Cdk5 regulates intracellular Ca^2+^ dynamics and Ca^2+^-mediated cell proliferation. We demonstrate that Cdk5 targets IP3R1 to control ER Ca^2+^ release and [Ca^2+^]_cyt_. These Cdk5-mediated Ca^2+^ dynamics are reflected in the disrupted Ca^2+^-mediated proliferation of *Cdk5*^−/−^ MEFs and development of *Cdk5*^−/−^ embryos.

## Materials and methods

### Materials

Dulbecco’s modified eagle’s medium (DMEM), heat-inactivated fetal bovine serum (FBS), EDTA-Trypsin, antibiotic–antimycotic, H_2_-DCFDA, MitoSOX red, MitoTracker green, Fluo-4 AM, Mag-Fluo-4 AM, ionomycin, GlutaMAX and an antibody against p27^*KIP1*^ (719,600) were from ThermoFisher Scientific (Waltham, MA). Mito-tempo and antibodies against Cdk5 (C-8), tubulin (D-10), IP3R1 (E-8), p21^*CIP1*^ (L-17), Prx1 (N-19) and actin (I-19) were from Santa Cruz Biotech (Manassas, VA, USA). The polyclonal antibodies against the two IP3R1 phosphopeptides, MLKIGTpS_421_VKEDKE and HVDRDPQEQVpT_799_PVK, were generated by GL Biochem. Ltd (Shanghai, China). The phosphoThr202/Tyr204-ERK1/2 antibody was from Cell Signaling (Danvers, MA, USA). The Ki67 (ab92353), Prx2 (ab109367), GAPDH (6C5) and Nrf2 (ab31163) antibodies and BAPTA-AM (ab120503) were from Abcam (Cambridge, MA, USA). The protease inhibitor cocktail, ATP and XeC were from Sigma (ON, Canada). Thapsigargin (TG) was a gift from Dr. Andrew Braun at the University of Calgary. The IP3R1 siRNAs were synthesized at the University of Calgary Core DNA Services. The peroxidase and serum-free protein block kits were from Dako (Glostrup, Denmark). The avidin and biotin block kit and DAB were from Zymed (CA, USA). The secondary antibodies were from Jackson ImmunoResearch Labs (Pennsylvania, USA). The Vectastain^®^ ABC Reagent was from Vector Laboratories (CA, USA). ECL reagent was from GE Healthcare (Chicago, USA). Jet prime transfection reagent was from Polyplus transfection (NY, USA).

### Animals

The *Cdk5*^*−/−*^ embryos that we used in our studies were generated by intercrossing *Cdk5*^*+/−*^ mice (Jackson Laboratory, Bar Harbor, ME, USA) maintained at the University of Calgary Animal Facility. Wt littermates were used as controls. All animal studies conformed to regulatory standards and were approved by the University of Calgary Health Sciences Animal Care Committee.

### Isolation and culture of primary MEFs

Primary MEFs were isolated from E13.5 Cdk5^+*/*+^ and Cdk5^*−/−*^embryos as described previously [34]. Briefly, embryos were washed with 1 × PBS, decapitated and eviscerated, and then washed again with PBS. Embryos were minced using sterile forceps and placed in 3–5 ml of 0.05% trypsin–EDTA, pipetted up and down to get cells into suspension and incubated at 37 °C for 5 min. Cell suspensions were transferred to tubes containing MEF medium (DMEM-high glucose supplemented with 10% FBS, 100 U/ml penicillin and 100 U/ml streptomycin, and 2 mM GlutaMAX) and then centrifuged at 1000 rpm for 5 min. Cell pellets were resuspended in fresh media and plated in 10 cm cell culture dishes. Primary MEFs were maintained in DMEM supplemented with 10% FBS, 50 U/ml penicillin and 50 mg/ml streptomycin under hypoxic condition (5% O_2_ and 5% CO_2_) at 37 °C. All experiments were performed using passage 2 to 7 (P2-P7) MEFs.

### Ca^2+^ measurement

(i) To measure resting [Ca^2+^]_cyt_, trypsinized 0.5 × 10^6^ MEFs were loaded with 5 µM Fluo-4 AM in DMEM for 1 h at room temperature. Cells were then washed three times with Ca^2+^-free EGTA-containing KRH buffer (25 mM HEPES, pH 7.4, containing 125 mM NaCl, 5 mM KCl, 1.2 mM MgCl_2_ and 6 mM glucose) and analyzed using a Shimadzu RF 5301PC spectrofluorometer. To minimize background fluorescence, 40 µM EDTA was added before reading the *F* values. *F*_max_ value was obtained after treatment with 0.02% saponin and addition of 2 µM CaCl_2_ three times (Supplementary Fig. 1A). *F*_min_ value was taken upon addition of 4 mM EDTA. [Ca^2+^]_cyt_ was calculated using the formula: free [Ca^2+^]_cyt_ = K_d_ [*F*–*F*_min_]/[*F*_max_–*F*] [44], whereas *K*_*d*_ (for Fluo-4) = 345 nM. (ii) To measure [Ca^2+^]_cyt_ transients by single-cell Ca^2+^ imaging, MEFs were seeded in 3.5 cm glass bottom petri dishes and stained with 5 µM Fluo-4 AM in HBSS (with 1.26 mM Ca^2+^) for 30 min at room temperature (RT). Cells were then washed three times with KRH buffer and analyzed by single-cell Ca^2+^ imaging using a Zeiss LSM 510 Meta confocal laser scanning microscope (20 × objective). Fluorescence signals were measured in 10–20 cells. Peak amplitudes were quantified as ratios of fluorescence (*F*/*F*_0_) after addition of ATP, XeC or TG. F_0_ represents basal fluorescence or fluorescence before stimulation. (iii) To measure ER Ca^2+^, MEFs were seeded in 3.5 cm glass bottom petri dishes and stained with 5 µM Mag-Fluo-4 AM in DMEM for 30 min at RT. Cells were then permeabilized with 0.1 mg/ml saponin for 45 s, washed with ICM buffer (10 mM HEPES, pH 7.4, containing 19 mM NaCl, 125 mM KCl, 1.5 mM Na_2_ATP, 0.735 mM MgCl_2_, 1 mM EGTA, 0.5 mM CaCl_2_) three times and analyzed using a Zeiss LSM 510 Meta confocal microscope (20 × objective). Fluorescence signals were measured in ten cells and quantified as ratios of fluorescence (F/F_0_) after addition of 500 nM IP3. F_0_ represents basal fluorescence or fluorescence before stimulation.

### siRNA transfection

MEFs (2.5 × 10^5^) seeded in 6 cm dishes were transfected with 100 nM IP3R1 siRNA #1 (AACATTGTGCAGAAAACAGCC) or #2 (AACAAAGAGATCCGTAGTAAG) for 48 h using Jet prime transfection reagent following the manufacturer’s protocol.

### Transfection of S_421_A and S_421_D IP3R1

pcDNA 3.0 carrying rat IP3R1 was obtained from Dr. I. Bezprozvanny at the University of Texas Southwestern Medical Center at Dallas. S_421_A IP3R1 and S_421_D IP3R1 were custom-generated by Genscript (USA). The IP3R also carries silent mutations: 2118 G > A, 2121 C > A, 2122 C > A, 2124 T > G, 2125 A > T and 2126 G > C that do not alter the IP3R1 amino acid sequence but confer resistance to IP3R1 siRNA #2. MEFs (5 × 10^5^) seeded in 6 cm dishes were co-transfected with IP3R1 siRNA (100 nM) and pcDNA 3.0 carrying S_421_A IP3R1 or S_421_D IP3R1 (2 µg) as per the Lonza nucleofector protocol (Basel Switzerland).

### ROS measurement

(i) For live-cell imaging, MEFs seeded in 4-chamber cover glass (Lab-Tek) were stained with 5 µM DCFDA or 200 nM MitoTracker green + 5 µM MitoSOX red to measure cytoplasmic hydrogen peroxide or mitochondrial superoxide levels, respectively. Images were taken using an Olympus 1X71 fluorescence microscope at 160 × magnification. (ii) By flow cytometry, MEFs (2.5 × 10^5^) seeded in 3.5 cm dishes were treated with 3 µM XeC, 10 µM ionomycin or 50 µM BAPTA-AM for 30 min. Cells were then washed with KRH buffer and harvested using trypsin. Cytoplasmic hydrogen peroxide and mitochondrial superoxide levels were measured by staining with 5 µM H_2_-DCFDA and 5 µM MitoSOX red, respectively, in KRH buffer for 30 min at 37 °C. Cells were then washed and resuspended in KRH buffer and analyzed by flow cytometry using a fluorescein isothiocyanate filter (530 nm) for DCFDA, a phycoerythrin filter (575 nm) for MitoSOX red.

### Proliferation analysis

1 × 10^3^ MEFs were seeded in 96 well plates (*n* = 3). *Cdk5*^*−/−*^ MEFs were treated (or untreated) with 3 µM XeC. After 1, 3 or 6 days in culture, cells were harvested using trypsin and stained with trypan blue, and viable cells were counted using a hemocytometer under an Olympus CK40 microscope.

### Immunoprecipitation and immunoblotting

Immunoprecipitation (IP) of clarified MEF lysates in lysis buffer (25 mM HEPES, pH 7.4, containing 250 mM NaCl, 1 mM PMSF, 1 mM EDTA, 1% Triton X-100, 1 µg/ml leupeptin, 1 µg/ml aprotinin, 1 µg/ml antipain and 15 µg/ml benzamidine) was performed using IP3R1 (E-8) antibody. IP samples or cell lysates were resolved by SDS–polyacrylamide gel electrophoresis (PAGE) and proteins were transferred onto nitrocellulose membranes, which were blocked in 5% skimmed milk and then incubated with the indicated primary antibody at 4 °C overnight. After washing with TBST buffer, containing 50 mM Tris–HCl, pH 7.6, 0.8% NaCl and 0.1% Tween-20, membranes were incubated with horseradish peroxidase-conjugated secondary antibody for 1 h. Immunoreactive bands were detected using ECL reagent (Amersham).

### Immunohistochemistry

E13.5 mouse embryos fixed in 4% paraformaldehyde (PFA) were sectioned to 10 µM thickness using a Leica RM2235 microtome. Tissue sections mounted on glass slides were incubated initially with peroxidase and then with avidin and biotin using a block kit followed by a serum-free protein block kit to eliminate non-specific binding of the primary antibody. The slides were then incubated with a Ki67 antibody at 1:100 dilution for 2 h, washed, and incubated with a secondary antibody for 40 min. Ki67-positive cells were detected by incubating with Vectastain^®^ ABC reagent for 30 min followed by DAB for 5 min. Tissue staining was visualized and photographed using an Olympus I ×71 light microscope with an attached Photometrics Coolsnap FX camera from Roper Scientific (Arizona, USA).

### Statistical analysis

Cdk5-regulated (i) [Ca^2+^]_cyt_ effect on ROS level, (ii) proliferation, and (iii) IP3-induced Ca^2+^ release were analyzed by one-way ANOVA. All other data were analyzed by Student’s t test. Significance was set at p < 0.05.

## Results

### Elevated ATP-evoked rise in [Ca^2+^]_cyt_ in *Cdk5*^−/−^ MEFs results from increased Ca^2+^ release from internal stores

**Fig. 1:**
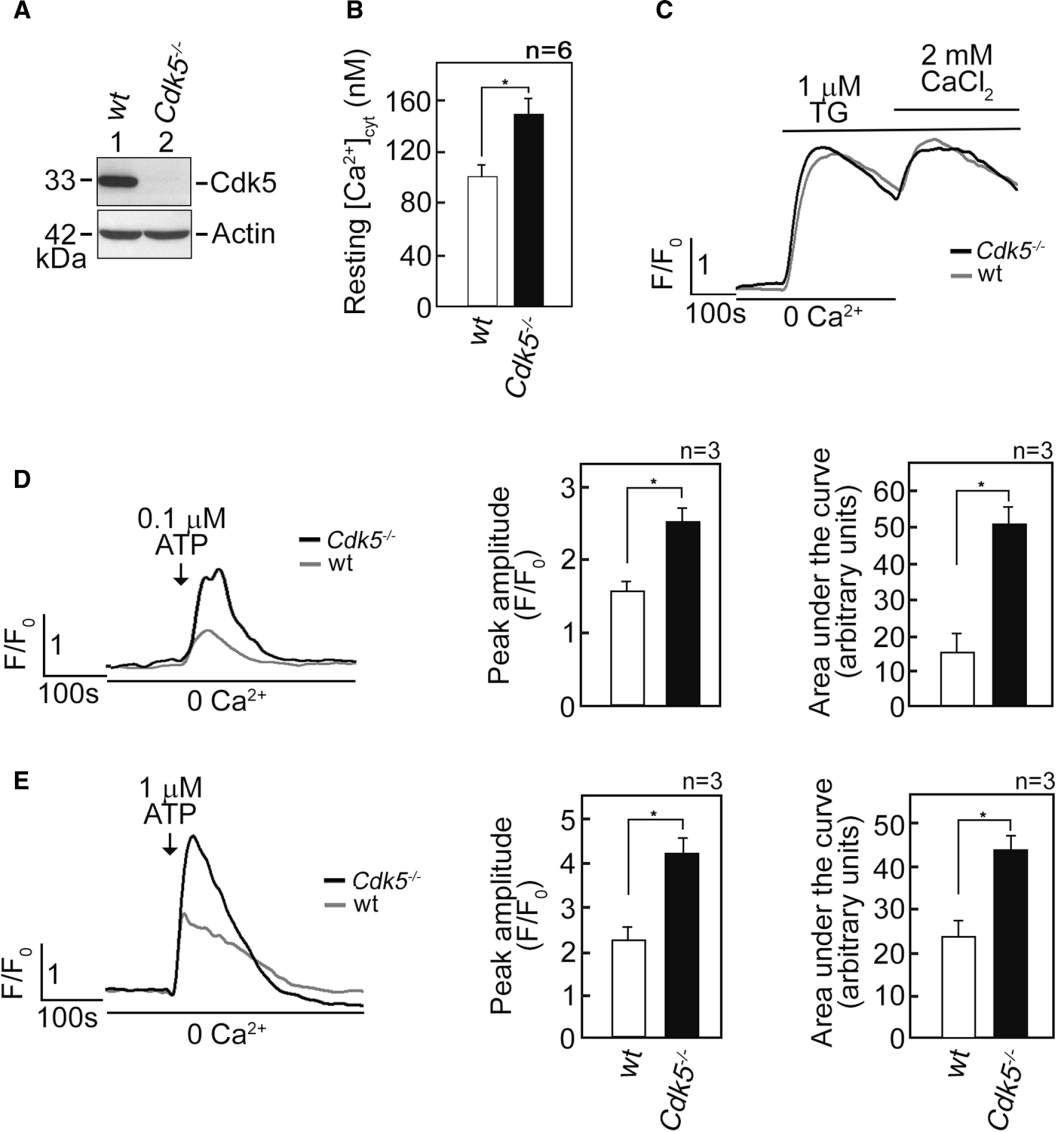
*Cdk5*^−/−^ MEFs exhibit increased [Ca^2+^]_cyt_ and increased ATP-induced Ca^2+^ release from internal stores. **A** Lysates of MEFs isolated from wt and *Cdk5*^−/−^ mouse embryos were analyzed by SDS-PAGE and immunoblotting for Cdk5. Actin blot was used as loading control. **B** Cdk5 loss caused an increase in free [Ca^2+^]_cyt_. Wt and *Cdk5*^−/−^ MEFs loaded with the cell-permeable intracellular Ca^2+^ indicator, Fluo-4 AM, were analyzed for [Ca^2+^]_cyt_ using spectrofluorometric Ca^2+^ imaging. Free [Ca^2+^]_cyt_ levels were measured as described in Materials and methods. Data represent means ± SEM from six independent experiments (*n* = 6). * indicates *p* < 0.01. **C** Wt and *Cdk5*^*−/−*^ MEFs loaded with Fluo-4 AM were analyzed for [Ca^2+^]_cyt_ transients following the addition of TG (1 µM) by single-cell Ca^2+^ imaging as described in Materials and methods. TG-induced Ca^2+^ release from internal stores corresponds to internal Ca^2+^ store capacity. After initial analysis in [Ca^2+^]-free buffer, capacitative Ca^2+^ entry from the extracellular milieu was determined upon addition of 2 mM CaCl_2_ in the presence of TG. Data represent means of Ca^2+^ signal traces from 15 cells. **D** and **E** Loss of Cdk5 caused an increase in ATP-induced Ca^2+^ release from internal stores. [Ca^2+^]_cyt_ in wt and *Cdk5*^*−/−*^ MEFs was measured following stimulation with 0.1 µM (**D**) or 1 µM (**E**) ATP in [Ca^2+^]-free buffer by single-cell Ca^2+^ imaging. Graphs in (**D**) and (**E**), right panels, represent peak amplitude values and integrated Ca^2+^ signals, which is the area under the curve (*AUC* area that begins immediately after addition of 0.1 mM ATP and ends when the Ca^2+^ trace goes back to the baseline level). Data represent means of Ca^2+^ signal traces from 20 cells. All values are means ± SEM from three independent experiments (*n* = 3). *p < 0.05

Our previous findings that Cdk5 regulates mitochondrial Ca^2+^ concentration ([Ca^2+^]_mt_) by controlling ER Ca^2+^ transfer to the mitochondria [34] led us to investigate whether Cdk5 also regulates free cytosolic Ca^2+^ concentration ([Ca^2+^]_cyt_). To do so, we initially performed spectrofluorometric analysis to measure resting [Ca^2+^]_cyt_ in Fluo-4 AM-loaded primary MEFs [34] isolated from wt and *Cdk5*^−/−^ mice embryos (Fig. 1A). As shown in Fig. 1B, the resting [Ca^2+^]_cyt_ in *Cdk5*^−/−^ MEFs was higher (*p* < 0.01) compared to that in wt MEFs (150 vs 100 nM), suggesting that loss of Cdk5 elicits a rise in free [Ca^2+^]_cyt_. We then examined three possible mechanisms that may have caused the rise in [Ca^2+^]_cyt_ in *Cdk5*^−/−^ MEFs: (i) reduced internal Ca^2+^ store capacity, (ii) increased Ca^2+^ influx from the extracellular milieu, and (iii) increased Ca^2+^ release from internal Ca^2+^ stores. We began by treating wt and *Cdk5*^−/−^ MEFs with thapsigargin (TG), a potent non-competitive inhibitor of the sarco/endoplasmic reticulum Ca^2+^ ATPase (SERCA) pump [45], that empties internal Ca^2+^ stores, allowing measurement of internal Ca^2+^ store capacity. This was followed by subjecting cells to external 2 mM CaCl_2_ to measure capacitative Ca^2+^ entry from the extracellular milieu. As shown in Fig. 1C, there was no difference in internal Ca^2+^ store capacity and capacitative external Ca^2+^ entry in wt and *Cdk5*^−/−^ MEFs. Since ATP, which binds cell surface purinergic receptors [46], induces Ca^2+^ release from internal stores [47] in Ca^2+^-free buffer, we examined whether increased [Ca^2+^]_cyt_ in *Cdk5*^−/−^ MEFs is due to internal store Ca^2+^ release by loading cells with Fluo-4 AM and treating them with ATP in Ca^2+^-free EDTA-containing buffer. By single-cell Ca^2+^ imaging analyses using a confocal laser scanning microscope, we found that 0.1 µM (Fig. 1D) and 1 µM (Fig. 1E) ATP caused greater [Ca^2+^]_cyt_ transients in *Cdk5*^−/−^ MEFs compared to wt, with greater rise in [Ca^2+^]_cyt_ transient at 1 µM ATP. These findings suggest that elevated ATP-evoked rise in [Ca^2+^]_cyt_ in *Cdk5*^−/−^ MEFs results from increased Ca^2+^ release from internal stores.

### Elevated ATP-evoked rise in [Ca^2+^]_cyt_ in *Cdk5*^−/−^ MEFs is due to increased Ca^2+^ release via IP3R channels

**Fig. 2:**
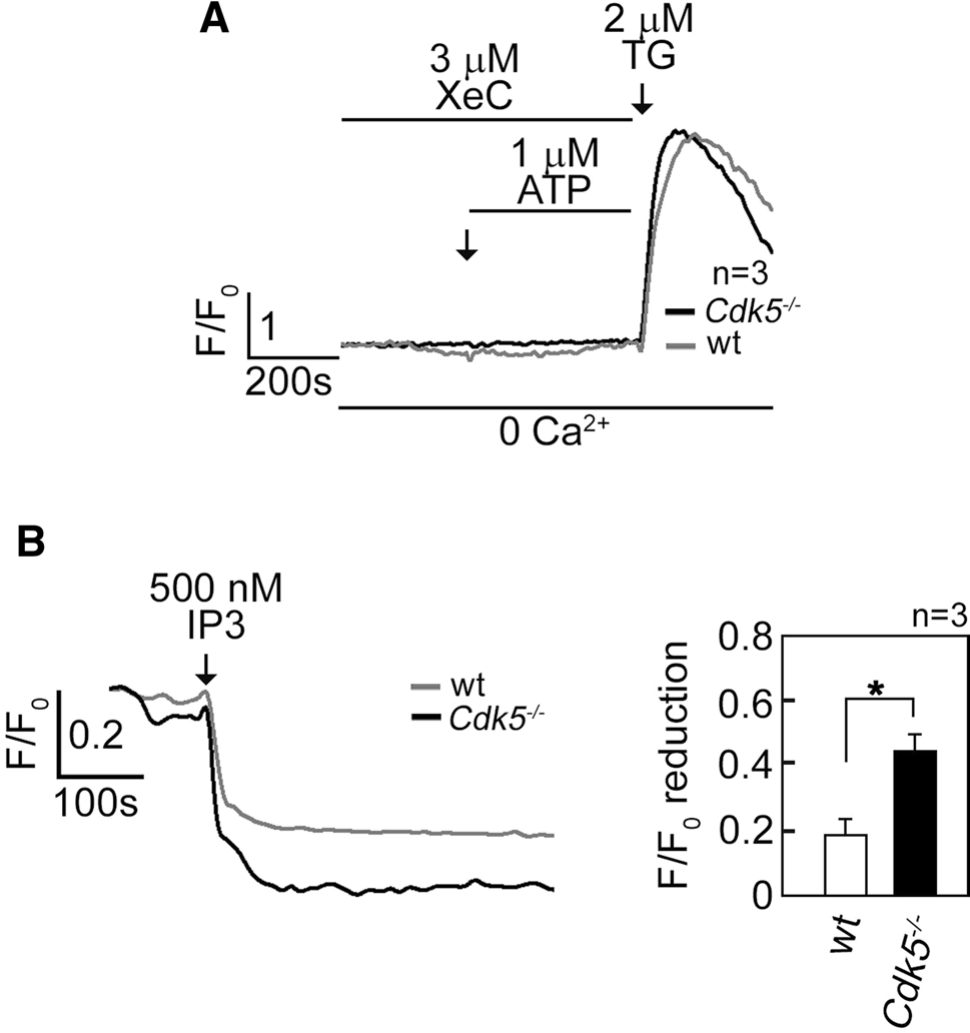
Increased [Ca^2+^]_cyt_ in *Cdk5*^−/−^ MEFs is due to increased IP3R-mediated Ca^2+^ release. **A** MEFs loaded with Fluo-4 AM and treated with 3 µM XeC followed by 1 µM ATP then 2 µM TG in Ca^2+^-free EGTA-containing KRH buffer were analyzed for [Ca^2+^]_cyt_ transients by single-cell Ca^2+^ imaging analyses. The similar increase in [Ca^2+^]_cyt_ in wt and *Cdk5*^−/−^ MEFs upon treatment with TG, which was added after 15 min of treatment with XeC, indicates comparable viability of these cells during analysis. Data represent means of Ca^2+^ signal traces from 15 cells. **B** Measurement of Ca^2+^ release from internal stores upon IP3 treatment is described in Materials and methods. Ca^2+^ release was measured every 4 s by single-cell Ca^2+^ imaging. Data represent means of Ca^2+^ signal traces from ten cells, and are results from one of three independent experiments showing similar patterns. Values are means ± SEM from the three separate experiments (*n* = 3). *p < 0.05

We next tested whether the ATP-induced rise in [Ca^2+^]_cyt_ in *Cdk5*^−/−^ MEFs occurs through IP3R. As shown in Fig. 2A, treatment of Fluo-4 AM-loaded and ATP-stimulated MEFs with xestospongin C (XeC) [48], a potent IP3R inhibitor, caused complete inhibition of the ATP-evoked [Ca^2+^]_cyt_ increase in both wt and *Cdk5*^−/−^ MEFs, indicating that such [Ca^2+^]_cyt_ increase is mediated by IP3R, which forms Ca^2+^ channels in the internal Ca^2+^ stores [47]. To examine whether loss of Cdk5 alters the IP3-mediated Ca^2+^ release from the ER, cells loaded with the ER Ca^2+^ probe, Mag-Fluo-4 AM [49], were treated with IP3 after permeabilization with saponin to facilitate IP3 access to IP3R. By single-cell Ca^2+^ imaging, we found that IP3 induced a greater decline in Mag-Fluo-4 signal in *Cdk5*^−/−^ MEFs compared to wt (Fig. 2B). Together, these results indicate that elevated ATP-evoked rise in [Ca^2+^]_cyt_ in *Cdk5*^−/−^ MEFs is due, at least in part, to increased ER Ca^2+^ release through IP3R channels.

### Cdk5 interaction with and phosphorylation of IP3R1 at S_421_ downregulate IP3R1-mediated ER Ca^2+^ release

**Fig. 3:**
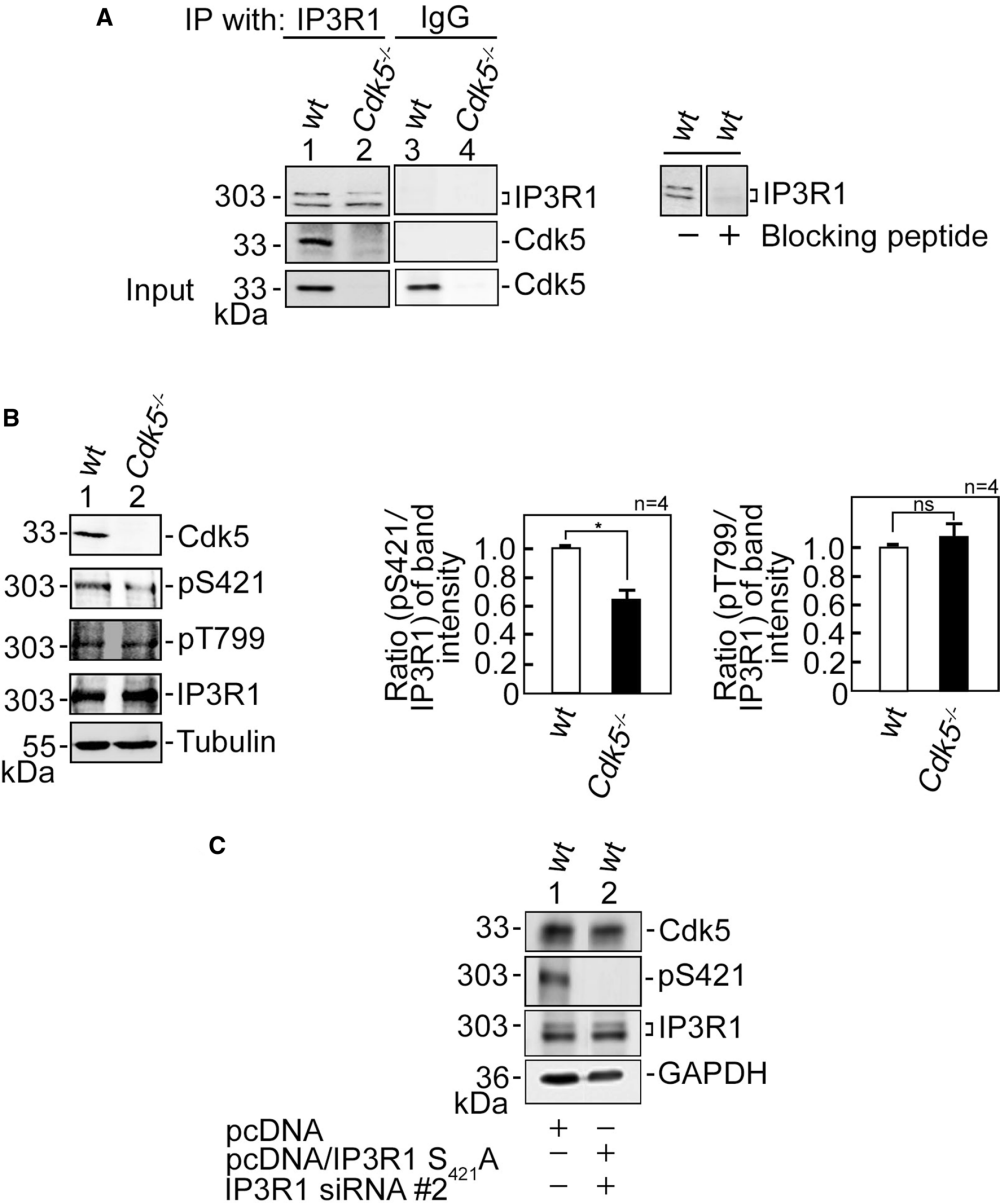
Cdk5 associates with and phosphorylates IP3R1 at Ser_421_. **A** Cdk5 associates with IP3R1. Lysates of wt and *Cdk5*^−/−^ MEFs were subjected to immunoprecipitation (IP) using IP3R1 antibody. The IPs were resolved by 4–20% gradient SDS-PAGE and then immunoblotted for IP3R1 and Cdk5 (left panel). To assess the specificity of the IP3R1 antibody, lysates of wt MEFs were blotted (right panel) with antibody blocked (lane 2) or not blocked (lane 1) with the peptide antigen that was used to raise the antibody. Lanes 3 and 4 represent IP control using normal IgG. **B** Cdk5 specifically phosphorylates IP3R1 at Ser_421_. Lysates of wt and *Cdk5*^−/−^ MEFs were subjected to SDS-PAGE and then immunoblotted for IP3R1 phosphoSer_421_ and phosphoThr_799_, IP3R1, Cdk5 and tubulin. Tubulin blot was used as loading control. Representative blots are from one of four independent experiments (*n* = 4) showing similar results. Ratios of levels of IP3R1 phosphoSer_421_ (middle panel) and phosphoThr_799_ (right panel) vs total IP3R were calculated following densitometric analysis of blots using NIH Image J 1.61. Standard deviations were calculated based on the ratios obtained from the four independent sets of experiments. Values from wt MEFs were normalized to 1.0. *p < 0.05. ns: not significant. **C** Shows the specificity of the IP3R1 phosphoSer_421_ antibody. Lysates of wt MEFs depleted of endogenous IP3R1, but expressing exogenous IP3R1 S_421_A (res) were subjected to immunoblotting for IP3R1 phosphoSer_421_, IP3R1, and Cdk5. GAPDH blot was used as loading control

We then sought to further investigate how ATP-evoked IP3R-mediated ER Ca^2+^ release increases in *Cdk5*^−/−^ MEFs. IP3R-mediated Ca^2+^ release is regulated by IP3R phosphorylation [18], and Cdk5, a Ser/Thr kinase with a (S/T)PX(K/H/R) preferred consensus phosphorylation site [3], localizes in MAMs [34] (Supplementary Fig. 3B) where it could interact with and phosphorylate IP3R. To test the possibility that Cdk5 associates with its likely IP3R isoform target, IP3R1, lysates of wt and *Cdk5*^−/−^ MEFs were subjected to immunoprecipitation (IP) using an IP3R antibody, and the IPs were immunoblotted for Cdk5. Co-IP of Cdk5 with IP3R1 (Fig. 3A) indicates interaction between the two proteins. However, co-transfection of Cdk5, p35 and IP3R1 in HEK293T cells followed by immunoprecipitation of Cdk5 or p35 showed the presence of the Cdk5/p35-IP3R1 complex. However, co-transfection of Cdk5 and IP3R1, but not p35, also showed Cdk5 interaction with IP3R1, indicating that such interaction does not require p35 (Supplementary Fig. 3C). We then examined whether Cdk5 phosphorylates its potential targets in IP3R1, S_421_PLK and T_799_PVK, by analyzing lysates of wt and *Cdk5*^−/−^ MEFs. Immunoblotting showed that while there was no difference in IP3R1 Thr_799_ phosphorylation in wt and *Cdk5*^−/−^ MEFs, IP3R1 Ser_421_ phosphorylation was reduced (*p* < 0.05) in *Cdk5*^−/−^ MEFs compared to wt (Fig. 3B), indicating that Cdk5 specifically targets Ser_421_ in IP3R1. The inability of IP3R1 Ser_421_ antibody to detect non-phosphorylatable IP3R1 S_421_A (Fig. 3C) confirms the specificity of the antibody.

**Fig. 4:**
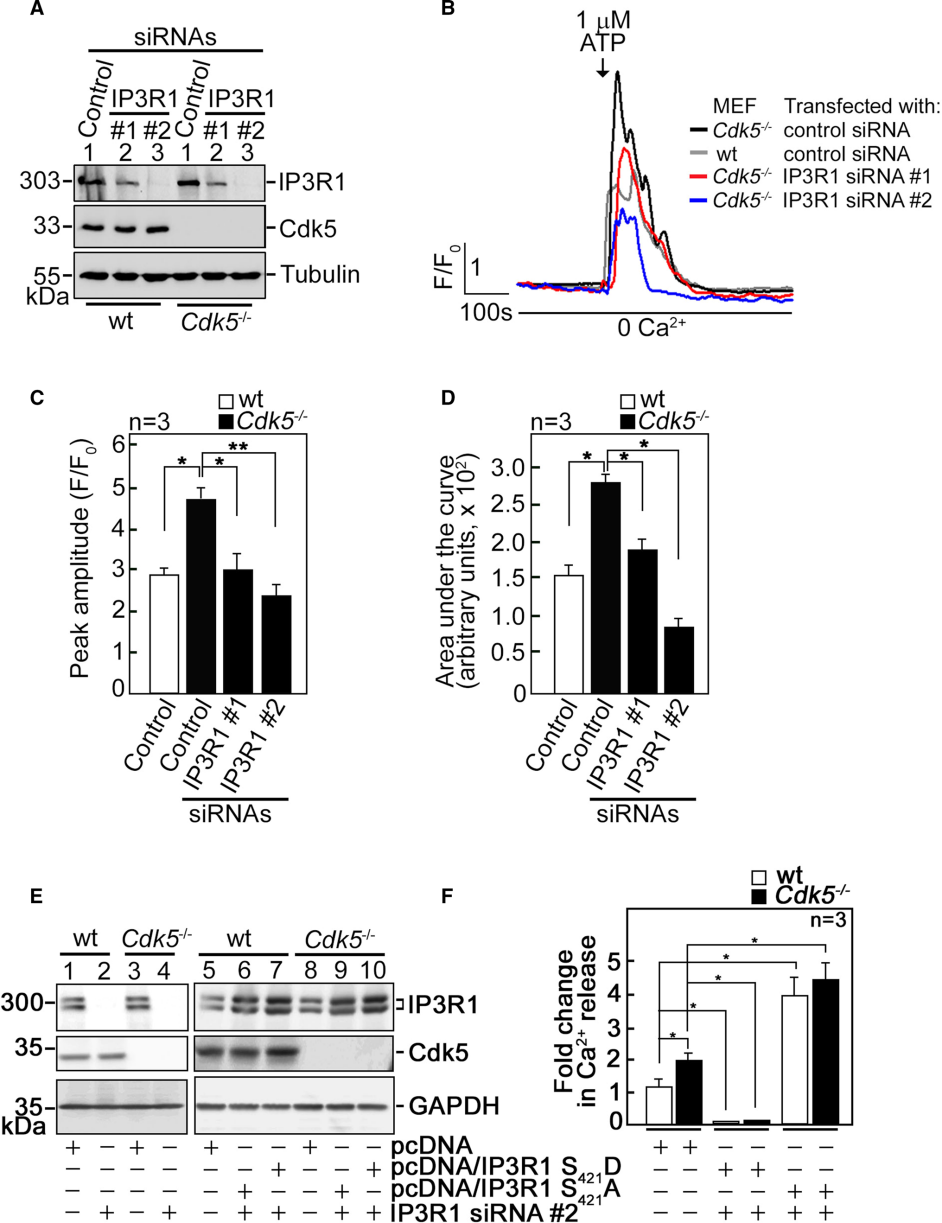
IP3R1 loss inhibits the ATP-induced increase in [Ca^2+^]_cyt_ in *Cdk5*^−/−^ MEFs. **A** Lysates of cells transfected with IP3R1 siRNA #1 or #2 for 48 h were resolved by SDS-PAGE and immunoblotting for IP3R1 and Cdk5. Tubulin blot was used to assess protein loading. Representative blots are from one of three independent experiments showing similar result are shown. **B** wt and *Cdk5*^−/−^ MEFs transfected with IP3R1 siRNA #1 or #2, loaded with Fluo-4 AM, and treated with 1 µM ATP were analyzed for [Ca^2+^]_cyt_ transients by single-cell Ca^2+^ imaging analyses in Ca^2+^-free buffer. Data are means of Ca^2+^ signal traces from 20 cells and are from one of three independent experiments showing similar results. [Ca^2+^]_cyt_ transients were further analyzed by measuring their peak amplitudes (**C**) and calculating the areas under the curve which begin immediately after addition of 1 mM ATP and ends when the Ca^2+^ trace goes back to the baseline level. **D** Values are means ± SEM from three independent experiments (*n* = 3). * and **Denote *p* < 0.05 and *p* < 0.01, respectively. **E** Wt and *Cdk5*^−/−^ MEFs were co-transfected with the indicated vector and IP3R1 siRNA #2. Cell lysates (40 µg) were resolved by SDS-PAGE and immunoblotted for IP3R1 and Cdk5. GAPDH blot was used as loading control. **F** Wt and *Cdk5*^−/−^ MEFs co-transfected with the indicated vector and siRNA #2 were loaded with Mag-Fluo-4 AM. ER Ca^2+^ release following IP3 treatment was measured by spectrofluorometry. Values, which represent the fold change in peak amplitudes, are means ± SEM from three independent experiments (*n* = 3). *p < 0.05

To further examine whether increased Ca^2+^ release in *Cdk5*^−/−^ MEFs is regulated by IP3R1, we utilized wt and *Cdk5*^−/−^ MEFs depleted of IP3R1 by siRNA #1 or #2 (Fig. 4A). We noted that siRNA #2 is more efficient at depleting IP3R1 compared to siRNA #1. IP3R1-depleted cells loaded with Fluo-4 AM then treated with 1 µM ATP in Ca^2+^-free buffer were analyzed by single-cell Ca^2+^ imaging. Consistent with our findings above, *Cdk5*^−/−^ MEFs showed increased ATP-evoked [Ca^2+^]_cyt_ transients compared to wt (Fig. 4B–D). Depletion of IP3R1 reversed the increase in [Ca^2+^]_cyt_ transients in *Cdk5*^−/−^ MEFs to levels close to those in wt MEFs. These [Ca^2+^]_cyt_ transients were quantified by measuring their peak amplitudes (Fig. 4C) and calculating the areas under the curve (AUC), which correspond to the integrated Ca^2+^ signals (Fig. 4D). These data imply that increased ATP-evoked ER Ca^2+^ release in *Cdk5*^−/−^ MEFs, as indicated by elevated ATP-evoked [Ca^2+^]_cyt_ transients in these cells, is mediated by IP3R1.

Next, we tested whether Cdk5-mediated phosphorylation of IP3R1 at S_421_ inhibits ER Ca^2+^ release. To do so, pcDNA carrying rat IP3R1, which shares 99.6% amino acid sequence identity with mouse IP3R1 (Supplementary Fig. 2), was subjected to site-directed mutagenesis to generate phosphomimetic IP3R1 S_421_D and non-phosphorylatable S_421_A, and for additional nucleotide substitutions to confer resistance to IP3R1 siRNA #2, but not alter the IP3R1 amino acid sequence (Supplementary Fig. 3A). Wt and *Cdk5*^−/−^ MEFs were then co-transfected with IP3R1 siRNA #2 (to deplete endogenous IP3R1) and pcDNA3.0 carrying IP3R1(res) S_421_D or S_421_A (Fig. 4E), loaded with Mag-Fluo-4 AM, and analyzed for IP3-induced ER Ca^2+^ release. As shown in Fig. 4F, *Cdk5*^−/−^ MEFs transfected with empty pcDNA3.0 displayed greater IP3-induced Ca^2+^ release than wt MEFs, which is consistent with our data in Fig. 2B. Expression of exogenous IP3R1 S_421_D caused complete inhibition of IP3-induced ER Ca^2+^ release in both wt and *Cdk5*^−/−^ MEFs depleted of endogenous IP3R1, while expression of exogenous IP3R1 S_421_A caused further increase in ER Ca^2+^ release compared to control vector-transfected cells. These findings and our earlier data, showing that Cdk5 loss, which reduces inhibitory phosphorylation of IP3R1 S_421_ (Fig. 3B), causes elevated IP3-induced Ca^2+^ release suggest that Cdk5 serves to downregulate ER Ca^2+^ release through inhibitory phosphorylation of IP3R1 at S_421_.

### Increased [Ca^2+^]_cyt_ due to Cdk5 loss induces ROS production, which upregulates Nrf2 level

**Fig. 5:**
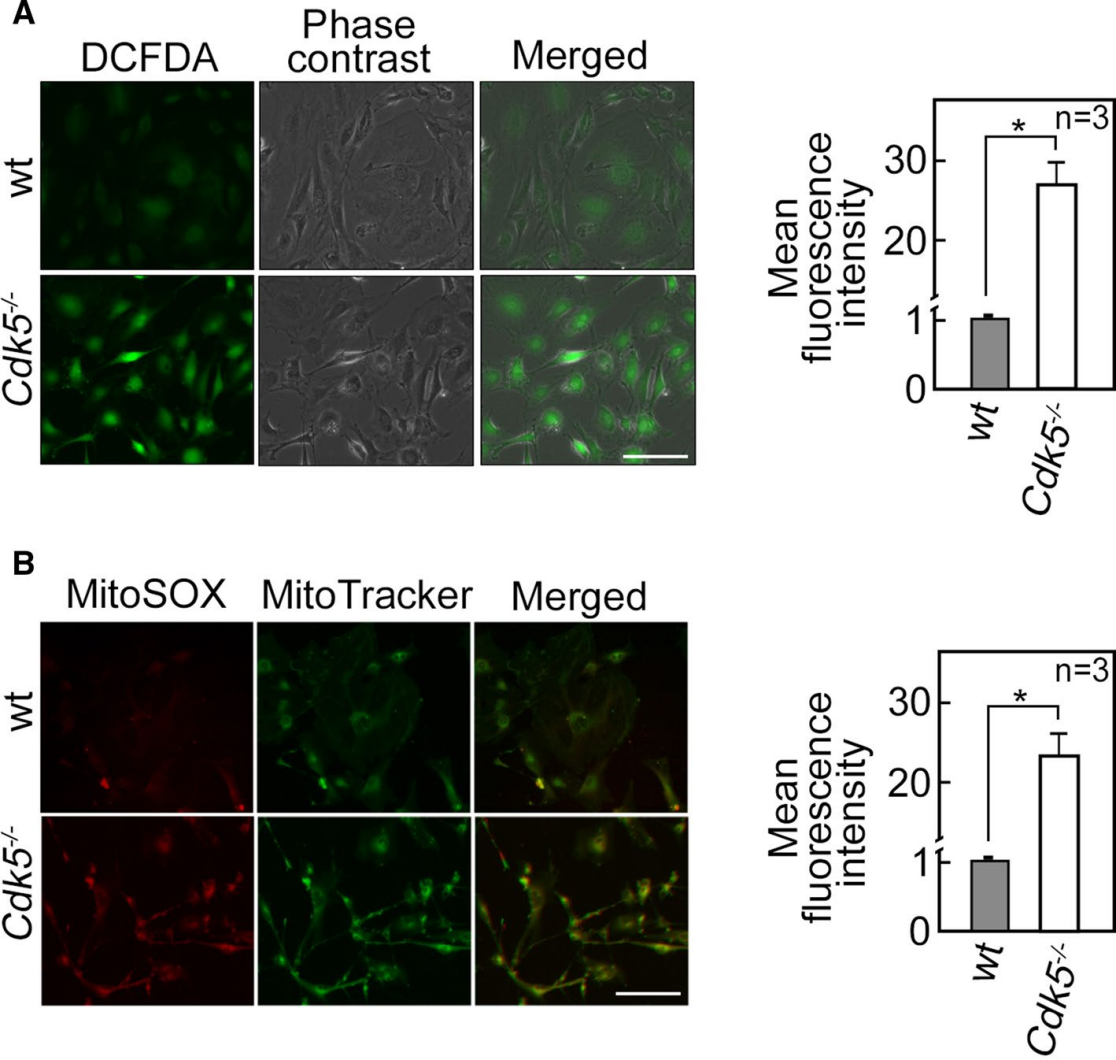
*Cdk5*^−/−^ MEFs exhibit increased ROS production. Wt and *Cdk5*^−/−^ MEFs stained with 5 µM DCFDA (**A**) or 5 µM MitoSOX red and 200 nM MitoTracker green (**B**) for 30 min were analyzed by live-cell imaging using an Olympus *I* × 71 fluorescence microscope at 160X magnification (left panels) and by flow cytometry (right panels). The left panels’ scale bars are equivalent to 100 µm. The right panels show the percentage increase in mean fluorescence intensity. Values from wt MEFs were normalized to 1.0. Values are means ± SEM from three independent experiments (*n* = 3). *p < 0.05

Since dysregulated [Ca^2+^]_cyt_ homeostasis affects intracellular ROS level [50, 51], and loss of Cdk5 alters [Ca^2+^]_cyt_, we next sought to assess intracellular ROS levels in wt and *Cdk5*^−/−^ MEFs. Cells stained with a fluorescent cytosolic ROS probe, 2',7'-dichlorodihydrofluorescein diacetate (DCFDA), were analyzed for intracellular hydrogen peroxide level by live-cell imaging using an Olympus 1X71 fluorescent microscope. As shown in Fig. 5A (left panel), *Cdk5*^−/−^ MEFs displayed increased DCFDA staining compared to wt. Consistent with the microscopic data, flow cytometry analysis showed an increase (*p* < 0.05) in intracellular hydrogen peroxide level in *Cdk5*^−/−^ MEFs compared to wt MEFs (Fig. 5A, right panel). Since mitochondria are a major source of ROS, we also examined ROS levels in this organelle by MitoSOX staining. Figure 5B (left panel) shows that mitochondrial superoxide anion levels were likewise higher in *Cdk5*^−/−^ MEFs compared with wt. Similarly, flow cytometry analysis showed an increase in mitochondrial superoxide anions in *Cdk5*^−/−^ MEFs compared to wt (Fig. 5B, right panel). These observations indicate that loss of Cdk5 induces ROS production.

**Fig. 6:**
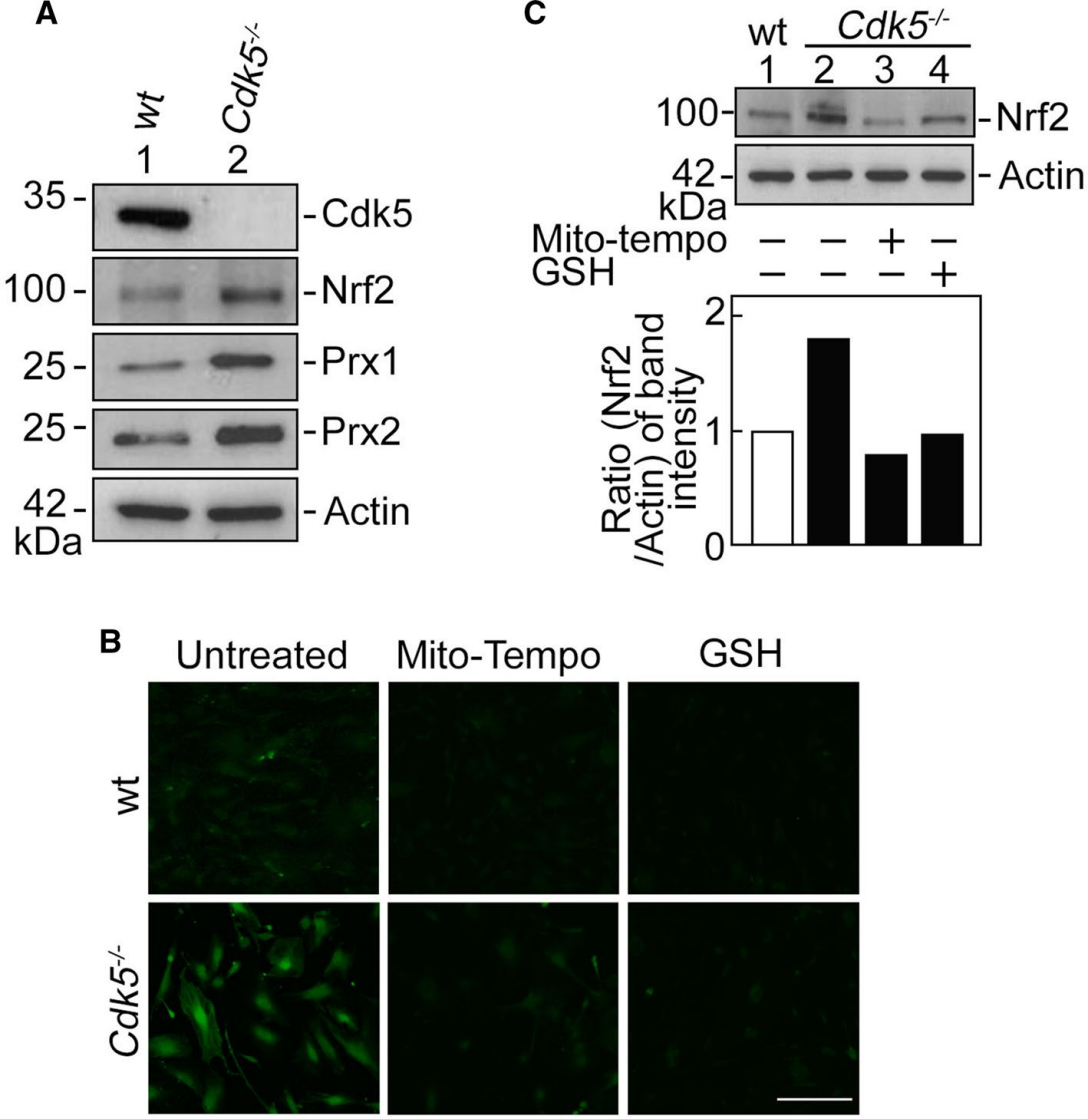
*Cdk5*^−/−^ MEFs exhibit increased Nrf2 level, and scavenging ROS with mito-tempo or GSH prevents the increase in ROS and Nrf2 level in these cells. **A** *Cdk5*^−/−^ MEFs show upregulated expression of Nrf2 and its downstream targets, Prx1 and Prx2. Lysates of wt and *Cdk5*^−/−^ MEFs were analyzed by SDS-PAGE and immunoblotting for Cdk5, Nrf2, Prx1 and Prx2. Actin blot was used to assess protein loading. **B** Wt and *Cdk5*^−/−^ MEFs treated with an ROS scavenger, mito-tempo (10 µM) or GSH (10 µM), and then stained with 5 µM DCFDA for 30 min were examined for cytoplasmic ROS level by live-cell imaging using an Olympus *I*×71 fluorescence microscope at 160 × magnification. Scale bar = 100 µm. **C** MEFs treated with mito-tempo or GSH were also analyzed by SDS-PAGE and immunoblotting for Nrf2. The graph (lower panel) shows the ratios of levels of Nrf2 vs actin calculated following densitometric analysis of blots using NIH Image J 1.61

ROS tightly regulates the activity of nuclear factor erythroid 2-related factor 2 (Nrf2) [52], which responds to oxidative stress by binding to the antioxidant response element (ARE) in the promoter of genes coding for antioxidant enzymes such as peroxiredoxin 1 and 2 (Prx1 and Prx2). Thus, we further examined the levels of Nrf2 in wt and *Cdk5*^*−/−*^ MEFs. Interestingly, Cdk5 loss, which induces ROS production, upregulates Nrf2 level as well as levels of the Nrf2 antioxidant protein targets, Prx1 and Prx2 (Fig. 6A). To establish a link between increased ROS level and upregulated Nrf2 level in *Cdk5*^−/−^ MEFs, cells treated with a ROS scavenger, mito-tempo or reduced glutathione (GSH) were (i) stained with DCFDA and analyzed for cytoplasmic ROS level by live-cell imaging, and (ii) subjected to SDS-PAGE and immunoblotting for Nrf2. As shown in Fig. 6B, and C, mito-tempo and GSH prevented the increase in ROS (Fig. 6B) and Nrf2 (Fig. 6C) levels in *Cdk5*^*−/−*^ MEFs. ROS and Nrf2 levels in these cells were reduced by the ROS scavengers to a level equivalent to that in untreated wt MEFs, indicating that increased ROS level in *Cdk5*^*−/−*^ MEFs upregulates Nrf2 level in these cells.

**Fig. 7:**
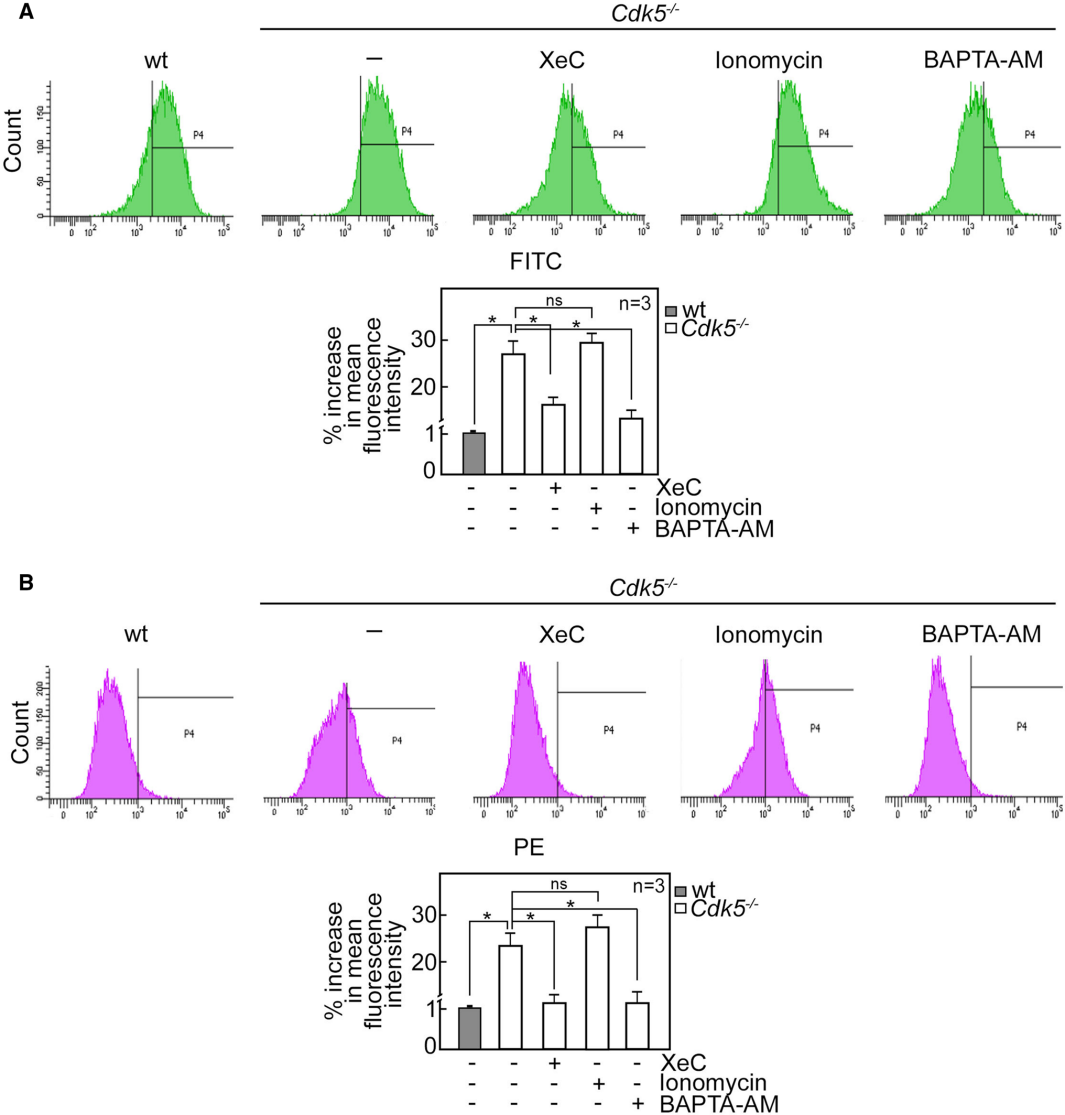
[Ca^2+^]_cyt_ regulates ROS production in *Cdk5*^−/−^ MEFs. Wt and *Cdk5*^−/−^ MEFs treated with 3 µM XeC, 10 µM ionomycin or 50 µM BAPTA-AM then stained with DCFDA (**A**) or mitoSOX red (**B**) were subjected to flow cytometry to measure cytoplasmic and mitochondrial ROS levels, respectively. Graphs (lower panels) show the % increase in mean fluorescence intensity. Values from wt MEFs were normalized to 1.0. Values are means ± SEM from three independent experiments (*n* = 3). *p < 0.05. ns: not significant, FITC: Fluorescein isothiocyanate, PE: Phycoerythrin

Our next step was to examine the effect of Cdk5-regulated [Ca^2+^]_cyt_ on ROS level. To do so, wt and *Cdk5*^−/−^ MEFs treated with XeC, ionomycin or BAPTA-AM and then stained with DCFDA (Fig. 7A) or mitoSOX red (Fig. 7B) were subjected to flow cytometry. As shown in Fig. 7A and B, inhibition of IP3R-mediated Ca^2+^ release with XeC and chelating Ca^2+^ with BAPTA-AM in *Cdk5*^−/−^ MEFs reduced ROS production to a level close to that in wt. As expected, treatment of *Cdk5*^−/−^ MEFs with the membrane permeable Ca^2+^ ionophore, ionomycin, increased ROS level. These findings indicate that increased [Ca^2+^]_cyt_ in *Cdk5*^−/−^ MEFs upregulates ROS production. We then examined the effect of scavenging ROS with GSH or mito-tempo on [Ca^2+^]_cyt_ in *Cdk5*^−/−^ MEFs. To do so, wt and *Cdk5*^−/−^ MEFs treated with GSH or mito-tempo were stained with Fluo-4 AM to measure [Ca^2+^]_cyt_ in these cells. As shown in Supplementary Fig. 4, GSH and mito-tempo had no effect on [Ca^2+^]_cyt_ in *Cdk5*^−/−^ MEFs under a condition where BAPTA-AM reduced [Ca^2+^]_cyt_ to a level similar to that in wt. These results indicate that while [Ca^2+^]_cyt_ regulates ROS production in *Cdk5*^−/−^ MEFs, ROS level does not influence [Ca^2+^]_cyt_ in these cells.

### Altered Ca^2+^ dynamics in *Cdk5*^−/−^ MEFs correspond to accelerated cell proliferation that correlates with increased body weight and size in *Cdk5*^*−/−*^ embryos

**Fig. 8:**
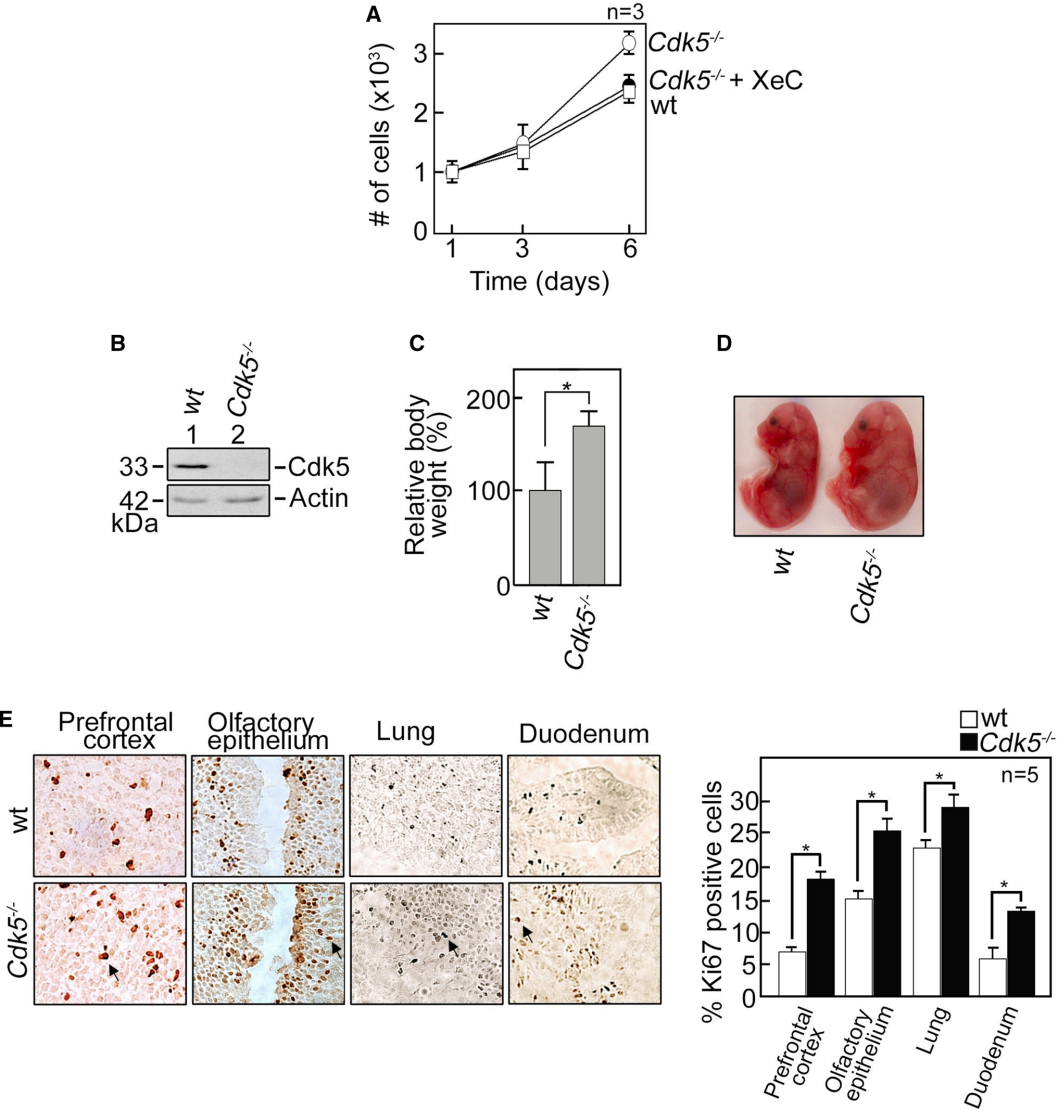
*Cdk5*^*−/−*^ MEFs show increased proliferation and *Cdk5*^*−/−*^ mouse embryos are heavier and bigger than wt. **A** *Cdk5*^*−/−*^ MEFs proliferate at a faster rate compared to wt, but treatment with XeC reverses this phenotype in *Cdk5*^−/−^ MEFs. Proliferation was measured as described in Materials and methods. **B** Homogenates (20 μg) of tails from E16.5 wt and *Cdk5*^−/−^ embryos were analyzed by SDS-PAGE and immunoblotting for Cdk5. Actin blot was used as loading control. **C** Body weights of E16.5 wt (*n* = 4) and *Cdk5*^−/−^ (*n* = 5) embryos from four litters were measured. *p < 0.05. **D** Representative images of E16.5 wt and *Cdk5*^−/−^ littermates. **E** Immunohistochemistry of prefrontal cortex, olfactory epithelium, lung and duodenum from wt and *Cdk5*^−/−^ littermates. Embryos were sectioned to 10 μm thickness and stained for Ki67. Arrows are directed at Ki67-positive cells. The graph showing the percentage of Ki67-positive cells was calculated from five non-overlapping fields (*n* = 5). *p < 0.05

IP3R-mediated Ca^2+^ transients regulate G_1_/S transition during cell-cycle progression [53] and cell proliferation [18, 19], and Cdk5, which regulates intracellular Ca^2+^ dynamics, has been implicated in cell proliferation [7, 8]. This prompted us to test whether altered Ca^2+^ dynamics in ex vivo* Cdk5*^−/−^ MEFs is associated with proliferation defect. As shown in Fig. 8A, *Cdk5*^*−/−*^ MEFs proliferate at a faster rate compared to wt, indicating that Cdk5 loss, which triggers a rise in [Ca^2+^]_cyt_, accelerates proliferation in MEFs. To establish a link between altered Ca^2+^ dynamics and increased proliferation in *Cdk5*^−/−^ MEFs, proliferation of cells treated with XeC was examined. As shown in Fig. 8A, treatment with XeC reversed the increase in *Cdk5*^*−/−*^ MEF proliferation to a level equivalent to that in wt, indicating that accelerated proliferation in *Cdk5*^−/−^ MEFs is linked to altered IP3R-mediated Ca^2+^ dynamics. We then tested whether the proliferation error in *Cdk5*^*−/−*^ MEFs is recapitulated in mice. Since *Cdk5*^*−/−*^ mice exhibit perinatal mortality (i.e., 64% die in utero and newborns are either dead or weak and die within 12 h after birth) [54], we isolated embryonic day 16.5 (E16.5) *Cdk5*^+*/*+^ and *Cdk5*^*−/−*^ embryos (Fig. 8B) from pregnant *Cdk5*^+*/*–^ mice, and body weights and sizes were compared. As shown in Fig. 8C, the *Cdk5*^*−/−*^ embryos weighed more (*p* < 0.05) than their wt littermates. Figure 8D shows representative wt and *Cdk5*^*−/−*^ littermate embryos with the *Cdk5*^*−/−*^ embryo clearly bigger than the wt. By immunohistochemistry, we found increased staining for Ki67, a proliferation marker, in the prefrontal cortex, olfactory epithelium, lung, and duodenum of *Cdk5*^*−/−*^ embryos compared to wt (Fig. 8E), which likely accounts for their increased body weight and size. In addition, *Cdk5*^*−/−*^ MEFs have increased phospho-ERK1/2 but reduced p27^*KIP1*^ and p21^*CIP1*^ compared to wt (Supplementary Fig. 5). Taken together, our results indicate that altered Ca^2+^ dynamics due to Cdk5 loss correspond to accelerated cell proliferation that correlates with increased body weight and size in *Cdk5*^*−/−*^ embryos.

## Discussion

**Fig. 9:**
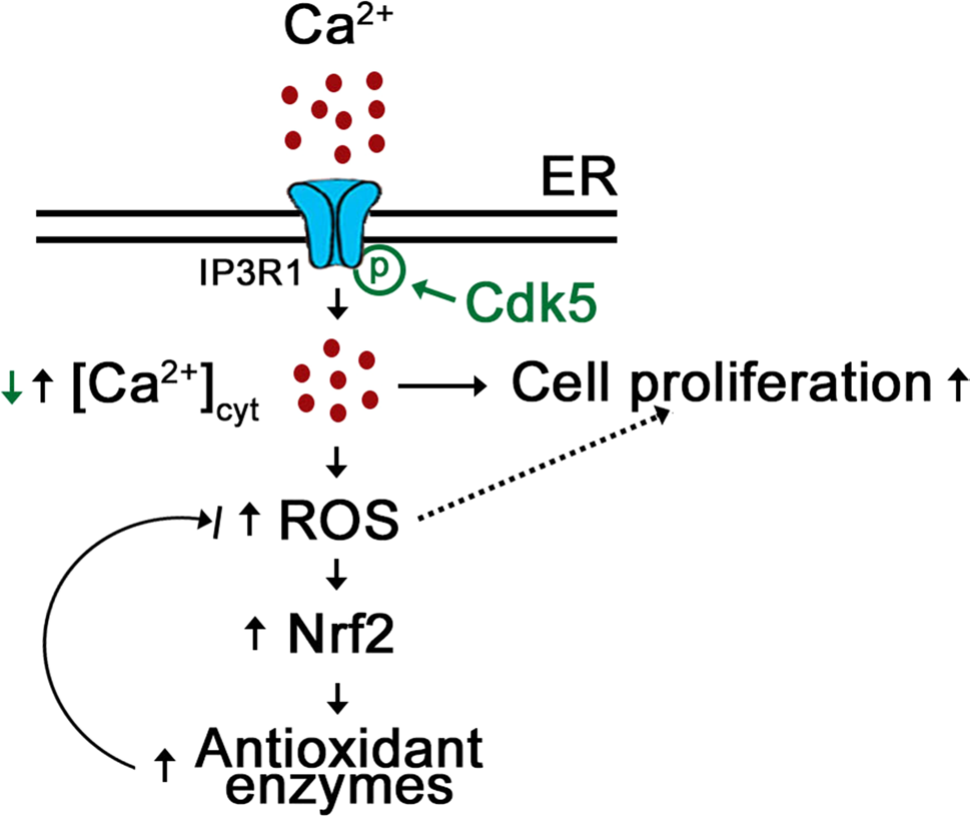
Proposed model illustrating how Cdk5 phosphorylation of IP3R1 (Ser_421_) controls IP3R1-mediated internal Ca^2+^ release and [Ca^2+^]_cyt_ (green text and arrow) and how loss of Cdk5 in *Cdk5*^*−/−*^ MEFs affects [Ca^2+^]_cyt_ and Ca^2+^-mediated processes (black text and arrows). Loss of Cdk5 reduces the phosphorylation of IP3R1 Ser_421_, causing increased IP3R1-mediated Ca^2+^ release. Subsequent rise in [Ca^2+^]_cyt_ increases ROS production, causing increased Nrf2 expression and activity, and increased expression of the NRF2 antioxidant targets such as Prx1 and Prx2. Adequate [Ca^2+^]_cyt_ permits progression of Ca^2+^-mediated proliferation, but excess levels cause increased cell proliferation

Our previous finding that Cdk5 localizes in MAMs, and regulates [Ca^2+^]_mt_ by controlling ER Ca^2+^ transfer to the mitochondria [34], points to a role for Cdk5 in regulating intracellular Ca^2+^ dynamics. However, the involvement of Cdk5 in this process remains to be investigated. In this study, using *Cdk5*^*−/−*^ MEFs, we demonstrated that the IP3R1 Ca^2+^ channel is a downstream target of Cdk5, which interacts with and phosphorylates IP3R1 at Ser_421_, a target that lies in the IP3-binding site. As illustrated in our proposed model (Fig. 9), Cdk5 phosphorylation of IP3R1 Ser_421_ controls IP3R1-mediated internal Ca^2+^ release as loss of Cdk5 in MEFs, and thus, loss of IP3R1 Ser_421_ phosphorylation triggers an increase in IP3R1-mediated Ca^2+^ release from internal stores, resulting in elevated [Ca^2+^]_cyt_. This rise in [Ca^2+^]_cyt_ causes accelerated proliferation in *Cdk5*^*−/−*^ MEFs, which correlates with increased body weight and size in *Cdk5*^*−/−*^ mouse embryos.

While the rise in free [Ca^2+^]_cyt_ in *Cdk5*^−/−^ MEFs could be due to reduced internal Ca^2+^ store capacity or increased Ca^2+^ influx from the extracellular milieu, we did not observe either of these in *Cdk5*^−/−^ MEFs. To test the possibility that loss of Cdk5 perturbs Ca^2+^ release from the ER to cause increased [Ca^2+^]_cyt_, we took advantage of the fact that ATP mediates Ca^2+^ release from internal stores [47], and that an ER Ca^2+^ probe, Mag-Fluo-4 AM, may be utilized to measure ER Ca^2+^ release. Using this approach, we found greater ATP-induced ER Ca^2+^ release in *Cdk5*^−/−^ MEFs compared to wt. IP3R inhibition with XeC [55] completely blocks this ATP-evoked [Ca^2+^]_cyt_ increase in both wt and *Cdk5*^−/−^ MEFs, indicating that such [Ca^2+^]_cyt_ increase is mediated by the IP3R Ca^2+^ channels in the ER. Our finding that IP3 induces a greater decline in Mag-Fluo-4 signal in *Cdk5*^−/−^ MEFs compared to wt further supports our view that Cdk5 serves to control the IP3R-mediated ER Ca^2+^ release that leads to increased [Ca^2+^]_cyt_ in *Cdk5*^−/−^ MEFs.

Since IP3R-mediated Ca^2+^ release is regulated by IP3R phosphorylation [18], and Cdk5, which localizes in MAMs [34], has two potential phosphorylation target sites in IP3R1, S_421_PLK and T_799_PVK, that exist in the IP3-binding site, we examined whether Cdk5 interacts with and phosphorylates IP3R1. Indeed, we found that Cdk5 interacts with and specifically phosphorylates IP3R1 at Ser_421_. This was demonstrated by reduced IP3R1 Ser_421_ phosphorylation in *Cdk5*^−/−^ MEFs, which increases in ER Ca^2+^ release. The specificity of IP3R1 immunoreactivity was verified by the loss of detectable IP3R1 in wt MEFs when the IP3R1 antibody was blocked with the peptide antigen that was used to generate the antibody. The detection of two IP3R1 immunoreactive bands may reflect immunoreactivity with (i) both unphosphorylated and phosphorylated forms, (ii) different isoforms, or (iii) intact and degraded forms of the protein. Partial reduction (*p* < 0.05) of IP3R1 S_421_ phosphorylation in *Cdk5*^−/−^ MEFs compared to wt suggests the presence of at least one other IP3R1 S_421_ kinase. In fact, Cdk1 has been shown to phosphorylate IP3R1 S_421_ [37]. Although IP3R1 S_421_A substitution was shown to increase IP3 binding to IP3R1 [35], its effect on ER Ca^2+^ release has not been investigated. Our data show that in endogenous IP3R1-depleted cells, exogenous IP3R1 S_421_D (res) expression inhibits ER Ca^2+^ release, while IP3R1 S_421_A expression enhances ER Ca^2+^ release substantiate the importance of inhibitory phosphorylation of IP3R1 S_421_, which prevents Ca^2+^ release from internal stores. Apparently, Cdk5 plays a significant role in this process. Our findings support the idea that MAM-associated Cdk5 negatively regulates the opening of the IP3R1 Ca^2+^ channel through phosphorylation of IP3R1 Ser_421_, which, as indicated above, lies in the IP3-binding site. It is interesting that ERK1/2 phosphorylation of Ser_436_, which also lies in the IP3-binding site, inhibits the opening of the IP3R1 channel as well [39, 40]. Since a rise in [Ca^2+^]_cyt_ can activate Ca^2+^-induced Ca^2+^ release (CICR) [56] from the internal stores, elevated ER Ca^2+^ release in *Cdk5*^−/−^ MEFs may propagate Ca^2+^ signals to neighboring organelles, causing a further rise in [Ca^2+^]_cyt_.

In addition to triggering a rise in free [Ca^2+^]_cyt_, loss of Cdk5 in MEFs further induces ROS production. The ability of XeC and BAPTA-AM to reverse the increase in ROS level in *Cdk5*^−/−^ MEFs indicates that ROS production occurs downstream of the IP3R1-mediated increase in [Ca^2+^]_cyt_ in these cells. Interestingly, we found that increased ROS production in *Cdk5*^−/−^ MEFs is associated with increased proliferation. Since increased ROS also induces apoptosis, it is possible that ROS-associated upregulation of Nrf2 and its antioxidant protein targets, Prx1 and Prx2, in *Cdk5*^−/−^ MEFs acts in a feedback control loop, ensuring that the ROS level in these cells does not exceed the threshold level that triggers apoptosis. This notion indicates the adaptability of MEFs under increased oxidative stress condition.

Cdk5 and IP3R-mediated Ca^2+^ oscillations have been shown to regulate cell-cycle progression [6, 7, 10, 11, 53] and proliferation [12–19]. Thus, it is not surprising that *Cdk5*^*−/−*^ MEFs, which have elevated [Ca^2+^]_cyt_ through IP3R1, proliferate at a faster rate compared to wt. The ability of XeC to reverse the increase in *Cdk5*^*−/−*^ MEF proliferation to a level equivalent to that in wt supports our view that accelerated proliferation in *Cdk5*^−/−^ MEFs is linked to IP3R-mediated increase in [Ca^2+^]_cyt_. This is consistent with previous reports that Cdk5 plays an inhibitory role in the neuronal cell cycle [6, 10, 11]. In addition, reduced level of p27^*KIP1*^ in *Cdk5*^*−/−*^ MEFs is consistent with the fact that IP3R-mediated Ca^2+^ oscillations stimulate proliferation through downregulation of p27^*KIP1*^ [1, 53]. Proliferation error in *Cdk5*^*−/−*^ MEFs correlates with increased weight and size in E16.5 *Cdk5*^*−/−*^ embryos, and increased number of Ki67-positive cells in various embryonic tissues, including prefrontal cortex, olfactory epithelium, lung and duodenum. Although we observed the same trend in body weight and size in earlier E13.5 *Cdk5*^*−/−*^ embryos, we note that later E18.5 *Cdk5*^*−/−*^ embryos were lighter and smaller than their wt counterparts. This may be due to the development of other abnormalities in *Cdk5*^*−/−*^ embryos as they exhibit perinatal mortality. It is known that ~ 64% of *Cdk5*^*−/−*^ embryos die in utero and newborns are either dead or weak and die within 12 h after birth [54]. Nonetheless, increased body weight and size in E16.5 *Cdk5*^*−/−*^ embryos are consistent with reduced levels of the cell-cycle inhibitors, p21^*CIP1*^ and p27^*KIP1*^, and increased body weight in p27^*KIP1*^ knockout mice [57].

In summary, we provide evidence that Cdk5 controls intracellular Ca^2+^ dynamics through phosphorylation of IP3R1 at Ser_421_, and Ca^2+^-mediated cell proliferation as indicated by increased *Cdk5*^−/−^ MEF proliferation that correlates with increased body weight and size in *Cdk5*^*−/−*^ embryos.

## Supplementary Information

Below is the link to the electronic supplementary material.

Supplementary file1 (PDF 155 KB)

Supplementary file2 (JPG 308 KB)

Supplementary file3 (JPG 1249 KB)

Supplementary file4 (JPG 1260 KB)

Supplementary file5 (JPG 1319 KB)

Supplementary file6 (JPG 991 KB)

Supplementary file7 (JPG 480 KB)

Supplementary file8 (JPG 315 KB)

Supplementary file9 (JPG 404 KB)

## Data Availability

All data generated or analyzed during this study are included in this published article (and its supplementary information files).
