# Supplementary material for: Cdk5 regulates IP3R1-mediated Ca2+ dynamics and Ca2+-mediated cell proliferation
Source: Cell Mol Life Sci. 2022 Aug 24;79(9):495. doi: 10.1007/s00018-022-04515-8 (PMC9402492; doi:10.1007/s00018-022-04515-8)
Supplement: Supplementary file 1 — Supplementary file1 (PDF 155 KB) [file 18_2022_4515_MOESM1_ESM.pdf]

Supplementary Figure legend

Supplementary Figure 1. Resting $[Ca^{2+}]_{cyt}$ in wt and *Cdk5$^{-/-}$* MEFs was measured as described in Materials and Methods. F_{max} value was obtained after treatment with 0.02% saponin and addition of 2 µM $CaCl_2$ three times. F_{min} value was taken upon addition of 4 mM EDTA. RFU: relative fluorescence units.

Supplementary Figure 2. IP3R1 amino acid sequence alignment of rat (UniprotKB-P29994) and mouse (UniprotKB-P11881).

Supplementary Figure 3. (A) shows the IP3R1 siRNA #2 target sequence, the corresponding endogenous IP3R1 nucleotide (NM_010585.5) and amino acid (UniprotKB: P11881) sequences, and the nucleotide substitutions in IP3R1(res) that do not alter the IP3R1 amino acid sequence, but confer resistance to IP3R1 siRNA #2. (B) shows that Cdk5 and IP3R1 co-exist in MAMs purified from wt MEFs. Purified MAMs were immunoblotted for ER-MAM markers [fatty acid-CoA ligase, long-chain (FACL4) and glucose-regulated protein 78 kDa (GRP78)], a mitochondria-MAM marker [voltage dependent anion channel (VDAC)], a nucleus marker [proliferating cell nuclear antigen (PCNA)], and a cytoplasmic marker [lactate dehydrogenase (LDH)]. (C) shows the interaction of Cdk5, IP3R1 and p35 in HEK293T cells. Cells transfected with the indicated plasmids were subjected to immunoprecipitation (IP) using HA and p35 antibodies. The IPs were resolved in 4-20% gradient SDS-PAGE then immunoblotted for Cdk5, IP3R1, p35 and GAPDH.

Supplementary Figure 4. Scavenging ROS with GSH or mito-tempo does not affect $[Ca^{2+}]_{cyt}$ in *Cdk5$^{-/-}$* MEFs. Wt and *Cdk5$^{-/-}$* MEFs treated or untreated with 10 µM GSH, 10 µM mito-tempo or 50 µM BAPTA-AM were stained with Fluo-4 AM for 30 min then analysed for $[Ca^{2+}]_{cyt}$ by measuring fluorescence at 494/506 nm using a microplate reader. Values are means ± SEM from three independent experiments (n=3). *$p<0.05$. ns: not significant.

Supplementary Figure 5. *Cdk5$^{-/-}$* MEFs have increased levels of ERK1/2 phosphorylation but reduced levels of p21^{CIP1} and p27^{KIP1}. Lysates of wt and *Cdk5$^{-/-}$* MEFs were analyzed by SDS-PAGE and immunoblotting for ERK1/2 phosphoThr202/Tyr204, p27^{KIP1}, p21^{CIP1} and Cdk5. Actin blot was used to assess protein loading. Graphs show the ratios of levels of ERK1/2 phosphoThr202/Tyr204, p27^{KIP1} and p21^{CIP1} vs actin calculated following densitometric analysis of blots using NIH Image J 1.61.

Supplementary Materials and Methods

Ca^{2+} measurement (Supplementary Figure 4). Cells (1×10^4) seeded in black bottom 96-well plates and treated (or untreated) with 10 µM GSH, 10 µM mito-tempo or 50 µM BAPTA-AM for 16 hrs were stained with 5 µM Fluo-4 AM in HBSS (with 1.26 mM Ca^{2+}) for 30 min at 37 °C. Cells were then washed twice with HBSS (without Ca^{2+}) and analyzed using a fluorescence microplate reader at 494/506 nm.

Overexpression of tagged proteins (Supplementary Figure 3C). HEK293 cells ($1X\ 10^6$) seeded on 6 cm dishes were transfected with pCMV-HA-Cdk5 (5 µg), pcDNA-IP3R1 (5 µg) and pcDNA-YFP-p35 (5 µg) using Lipofectamine 2000 (Thermofisher Scientific) as per the manufacture's protocol.

Isolation of MAM fractions. MAM was isolated using Percoll density gradient centrifugation as described previously [34]. Briefly, MEFs (3×10^7 cells) were homogenized in 10 ml of IBcells-1 buffer containing 225 mM mannitol, 75 mM sucrose, 0.1 mM EGTA, and 30 mM Tris–HCl (pH 7.4). Homogenates (H) were centrifuged at 600 ×g for 5 min at 4 °C. The resulting supernatants were subjected to further centrifugation at 7,000 ×g for 10 min at 4 °C. Pellets were resuspended in 10 ml of IBcells-2 buffer containing 225 mM mannitol, 75 mM sucrose and 30 mM Tris-HCl (pH 7.4) followed by centrifugation at 7,000 ×g for 10 min at 4°C. Pellets were resuspended again in 10 ml of IBcells-2 buffer and centrifuged at 10,000 ×g for 10 min at 4°C. The resulting pellets (crude mitochondria) were resuspended in 2 ml of mitochondria resuspending buffer (MRB: 250 mM mannitol, 0.5 mM EGTA and 5 mM HEPES, pH 7.4). The crude mitochondria were then layered in 8 ml of Percoll medium [225 mM mannitol, 25 mM HEPES (pH 7.4), 1 mM EGTA and 30% Percoll] and 4 ml of MRB then centrifuged at 95,000 ×g for 30 min at 4 °C. The MAM fraction appears as a white band. The MAM fraction was resuspended in 14 ml of MRB buffer and centrifuged at 6,300 ×g for 10 min at 4 °C. The resulting supernatants (~14 ml) of the MAM fraction, designated as crude MAM (C-MAM), were further subjected to centrifugation at 100,000 ×g for 1 hr. Pellets containing pure MAM were each resuspended in 50 µl of MRB buffer.
