# Supplementary figures and images for: Cdk5 regulates IP3R1-mediated Ca2+ dynamics and Ca2+-mediated cell proliferation
Source: Cell Mol Life Sci. 2022 Aug 24;79(9):495. doi: 10.1007/s00018-022-04515-8 (PMC9402492; doi:10.1007/s00018-022-04515-8)

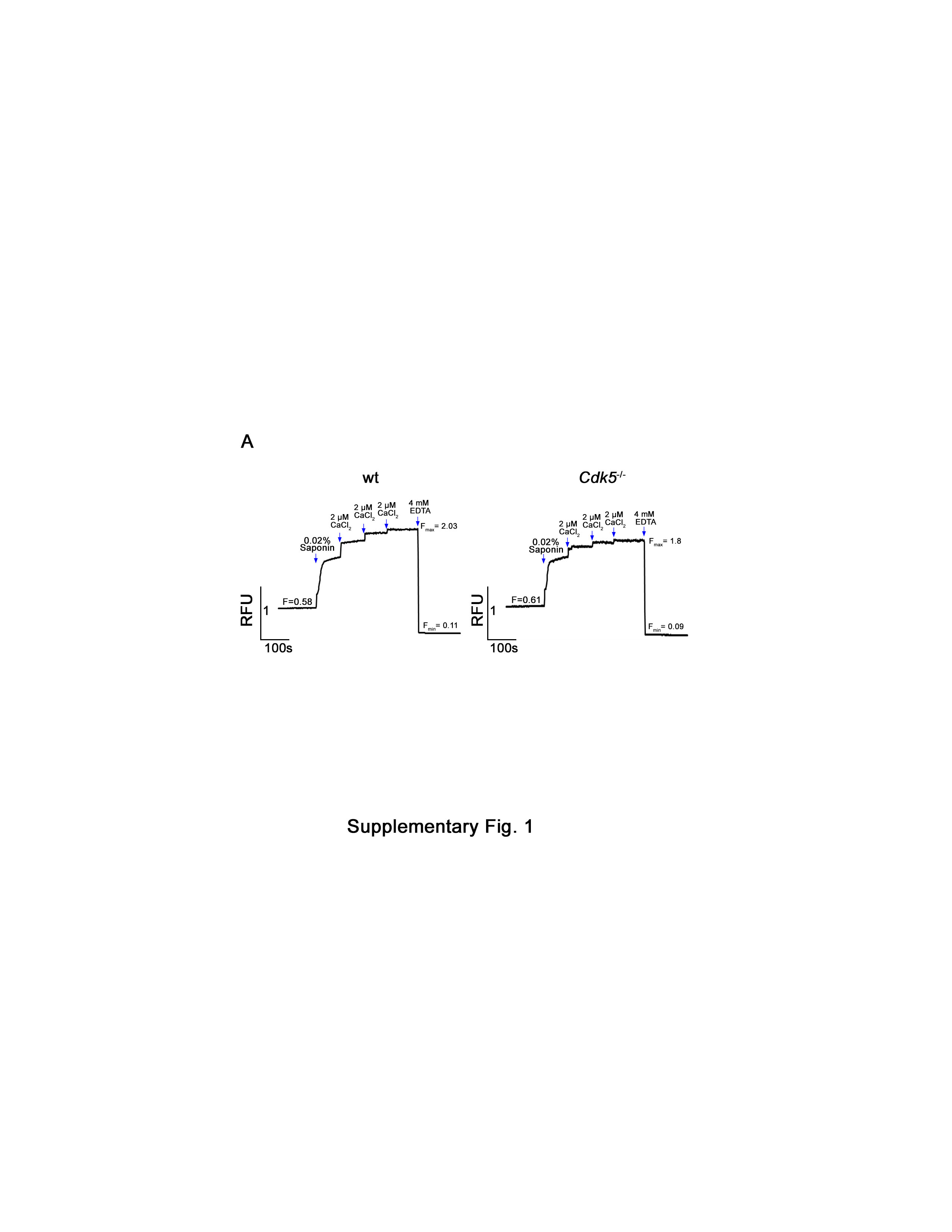

Supplement: Supplementary file 2 — Supplementary file2 (JPG 308 KB) [file 18_2022_4515_MOESM2_ESM.jpg]

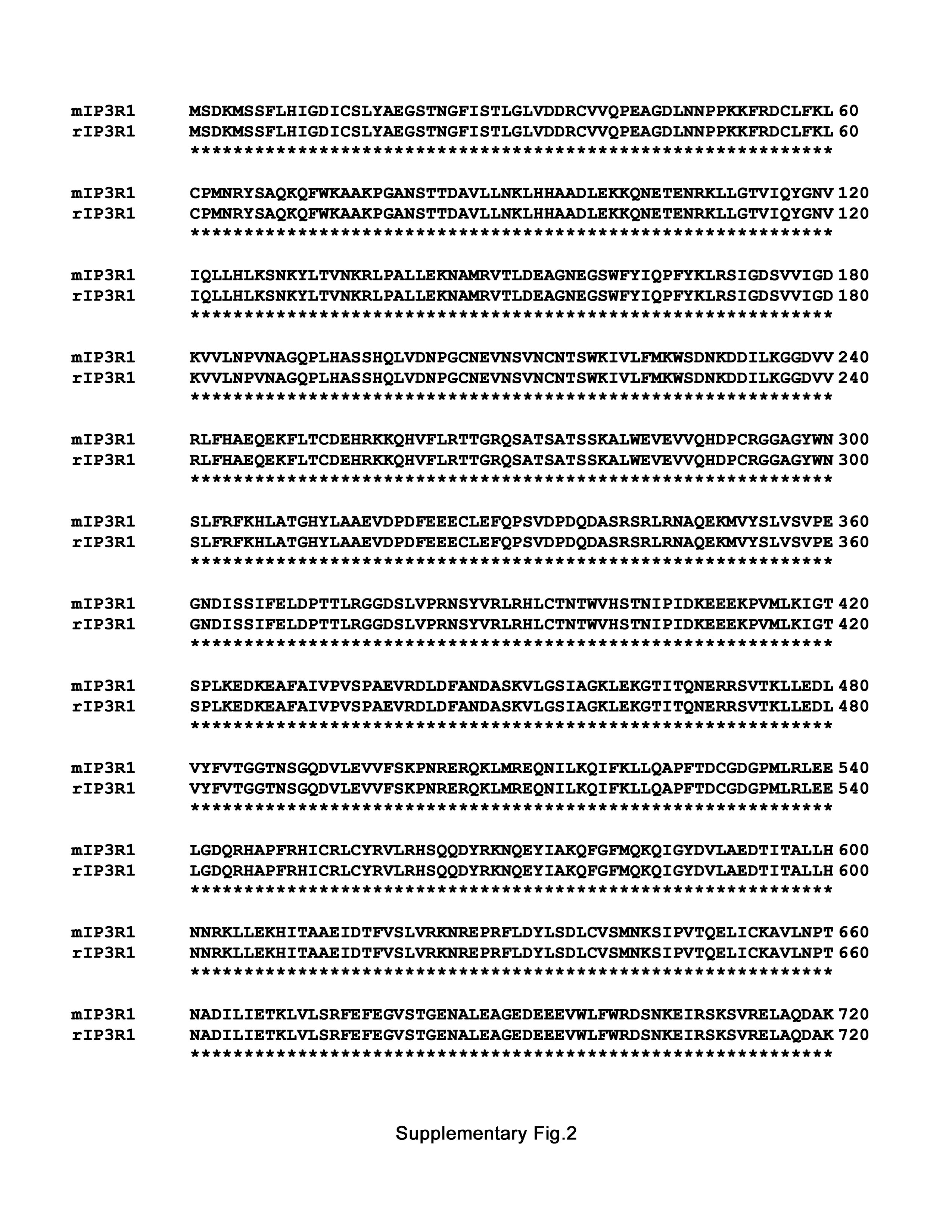

Supplement: Supplementary file 3 — Supplementary file3 (JPG 1249 KB) [file 18_2022_4515_MOESM3_ESM.jpg]

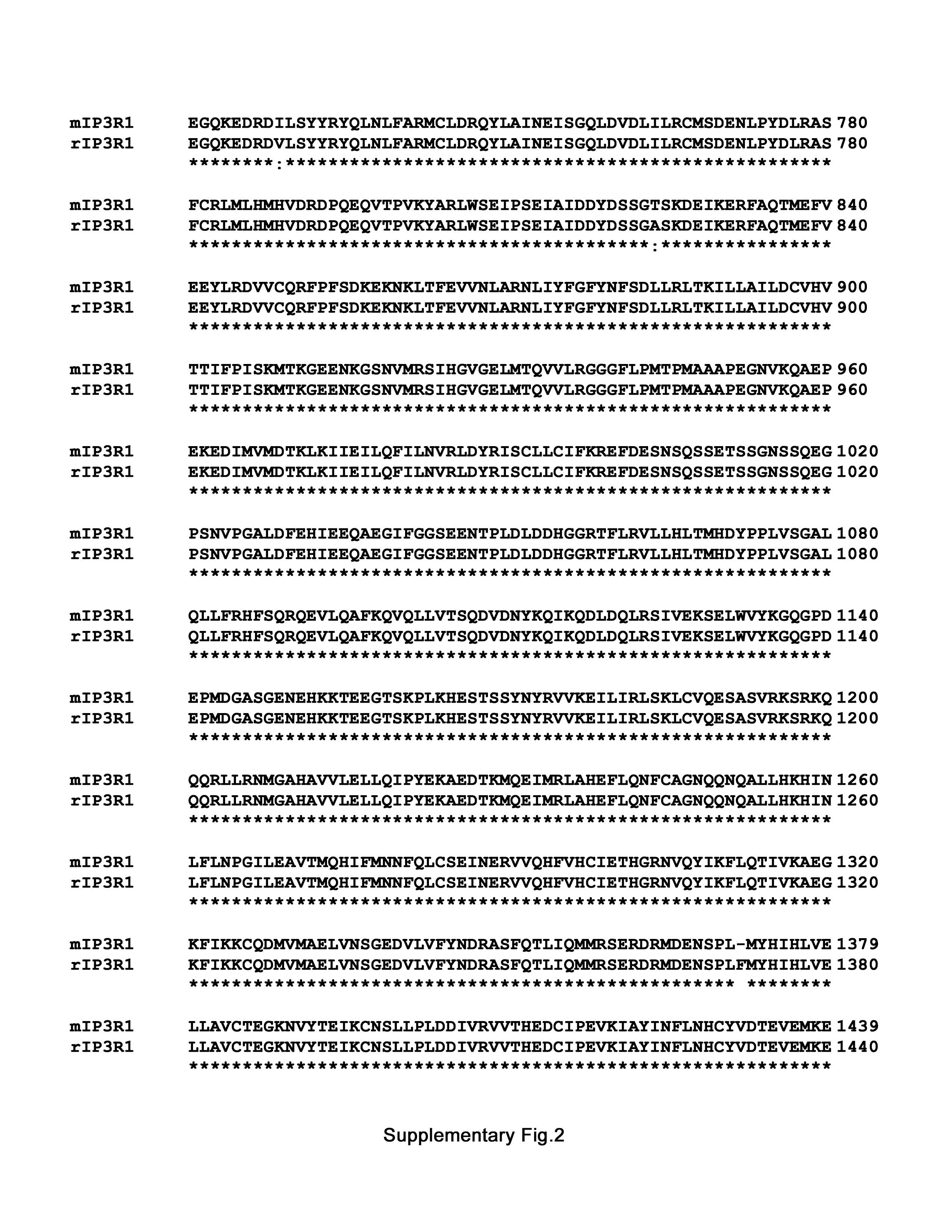

Supplement: Supplementary file 4 — Supplementary file4 (JPG 1260 KB) [file 18_2022_4515_MOESM4_ESM.jpg]

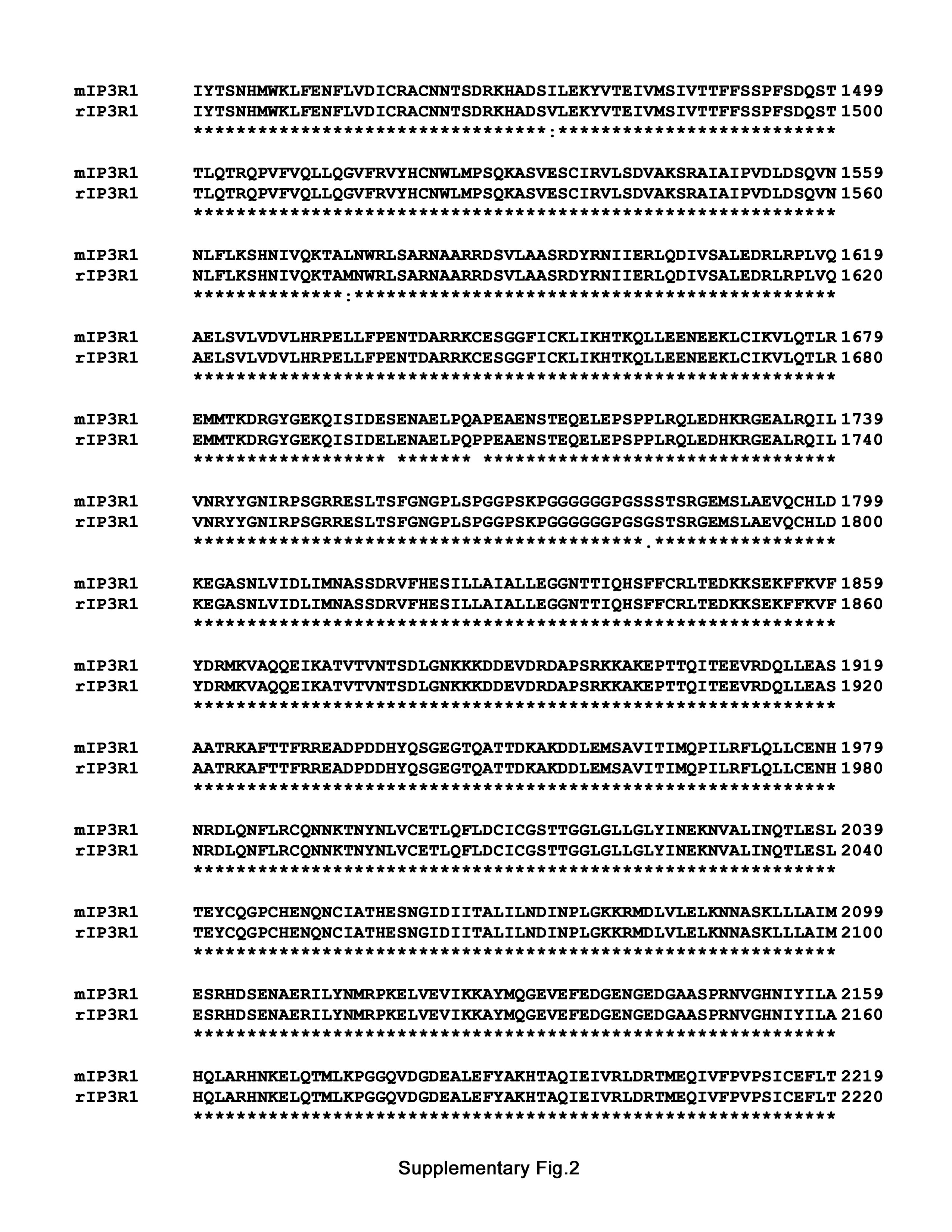

Supplement: Supplementary file 5 — Supplementary file5 (JPG 1319 KB) [file 18_2022_4515_MOESM5_ESM.jpg]

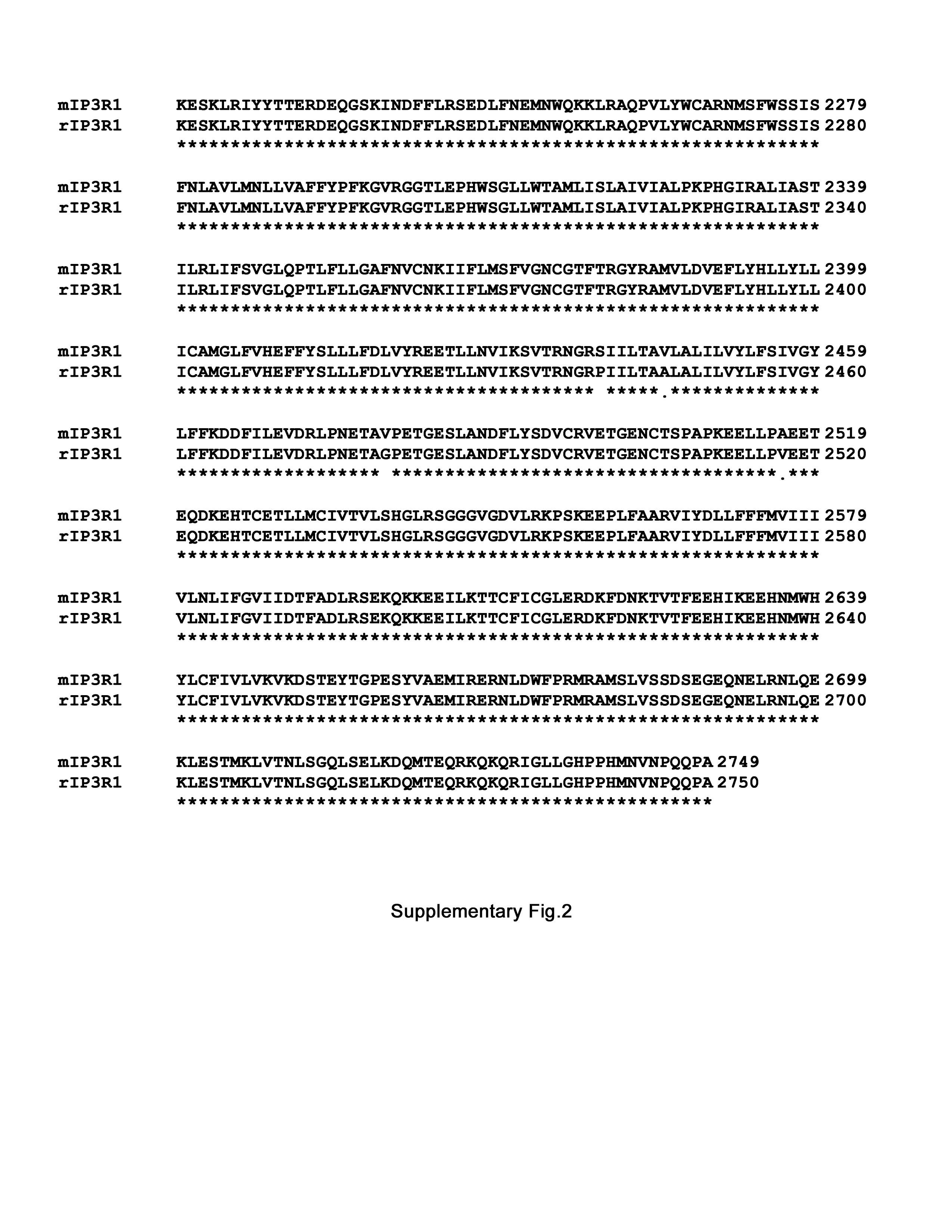

Supplement: Supplementary file 6 — Supplementary file6 (JPG 991 KB) [file 18_2022_4515_MOESM6_ESM.jpg]

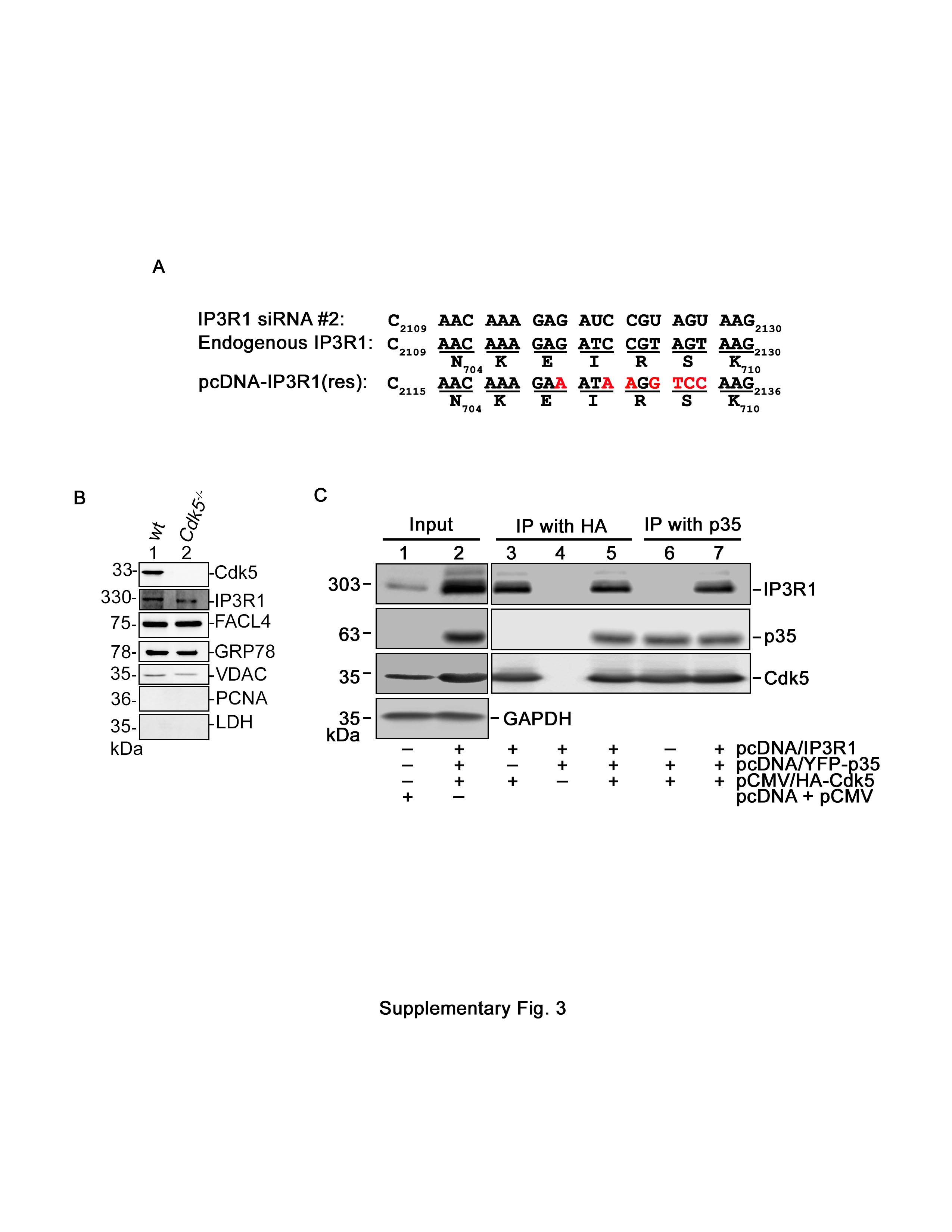

Supplement: Supplementary file 7 — Supplementary file7 (JPG 480 KB) [file 18_2022_4515_MOESM7_ESM.jpg]

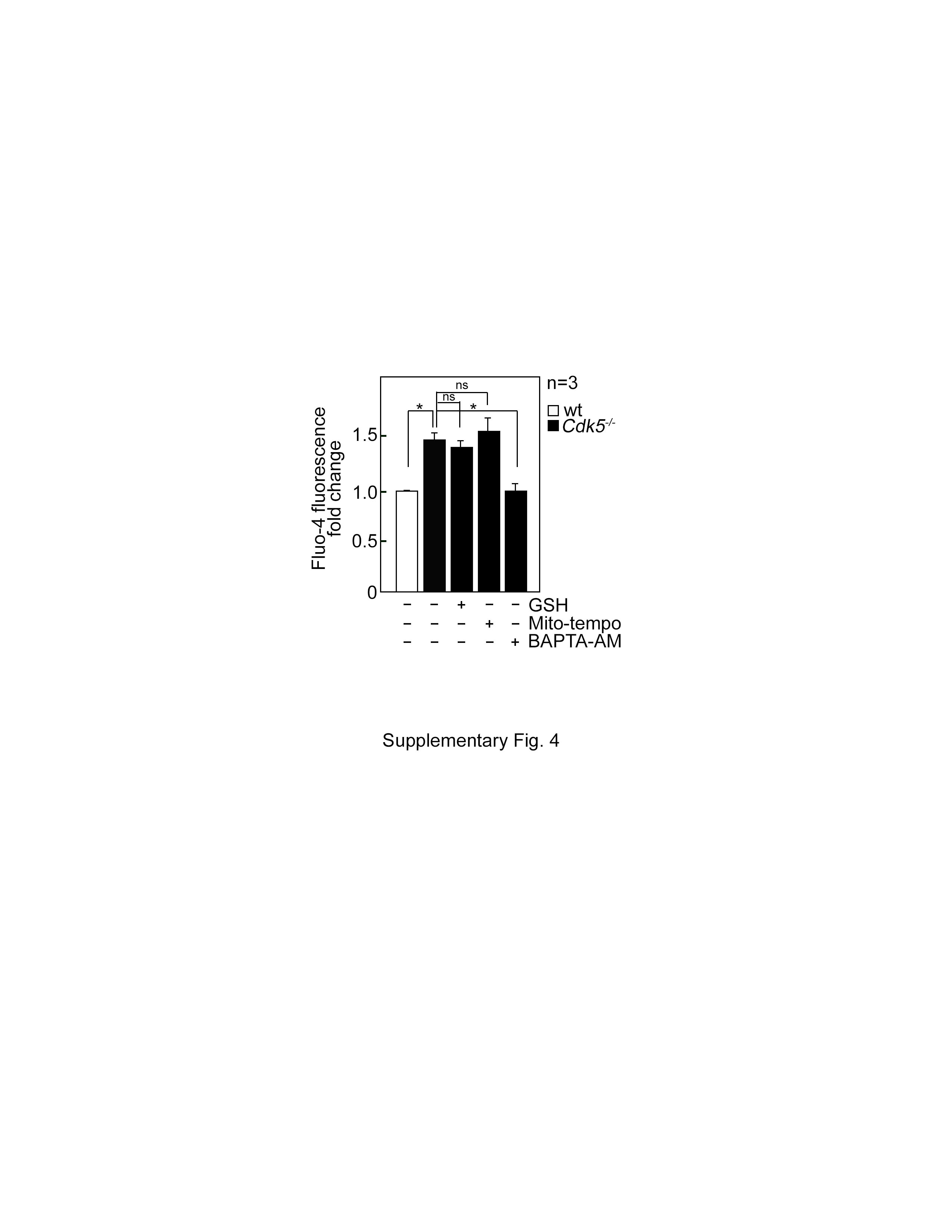

Supplement: Supplementary file 8 — Supplementary file8 (JPG 315 KB) [file 18_2022_4515_MOESM8_ESM.jpg]

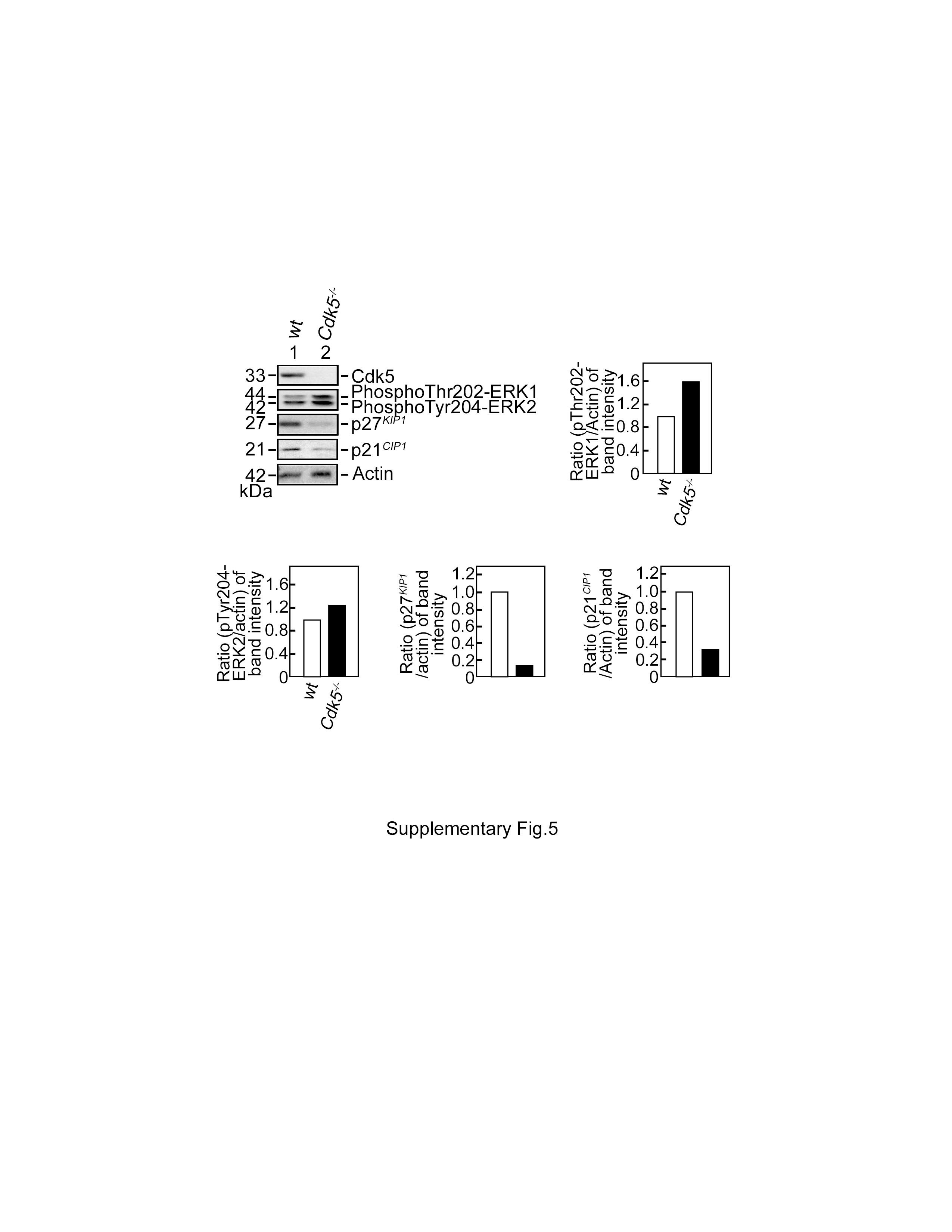

Supplement: Supplementary file 9 — Supplementary file9 (JPG 404 KB) [file 18_2022_4515_MOESM9_ESM.jpg]
